# Electric Field and Strain Tuning of 2D Semiconductor van der Waals Heterostructures for Tunnel Field-Effect Transistors

**DOI:** 10.1021/acsami.2c13151

**Published:** 2022-12-20

**Authors:** Konstantina Iordanidou, Richa Mitra, Naveen Shetty, Samuel Lara-Avila, Saroj Dash, Sergey Kubatkin, Julia Wiktor

**Affiliations:** † Department of Physics, Chalmers University of Technology, SE-412 96Gothenburg, Sweden; ‡ Department of Microtechnology and Nanoscience, Chalmers University of Technology, SE-412 96Gothenburg, Sweden

**Keywords:** 2D heterostructures, band alignment, electronic properties, external electric field, strain

## Abstract

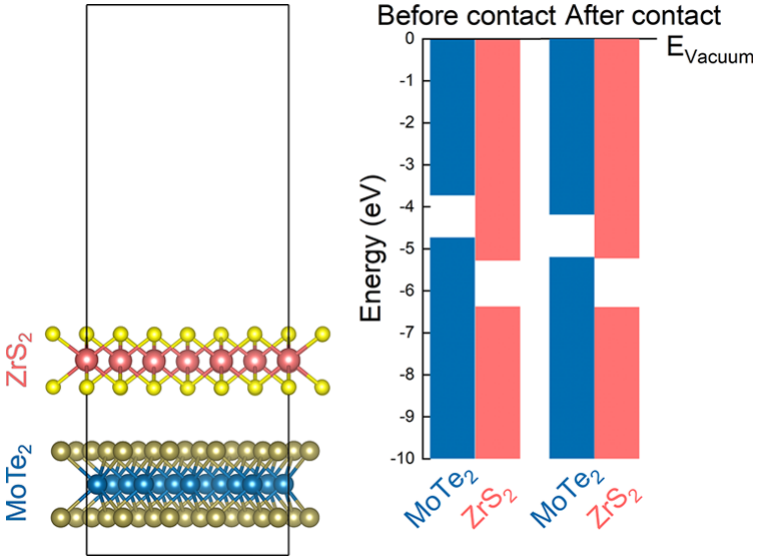

Heterostacks consisting of low-dimensional materials are attractive candidates for future electronic nanodevices in the post-silicon era. In this paper, using first-principles calculations based on density functional theory (DFT), we explore the structural and electronic properties of MoTe_2_/ZrS_2_ heterostructures with various stacking patterns and thicknesses. Our simulations show that the valence band (VB) edge of MoTe_2_ is almost aligned with the conduction band (CB) edge of ZrS_2_, and (MoTe_2_)_*m*_/(ZrS_2_)_*m*_ (*m* = 1, 2) heterostructures exhibit the long-sought broken gap band alignment, which is pivotal for realizing tunneling transistors. Electrons are found to spontaneously flow from MoTe_2_ to ZrS_2_, and the system resembles an ultrascaled parallel plate capacitor with an intrinsic electric field pointed from MoTe_2_ to ZrS_2_. The effects of strain and external electric fields on the electronic properties are also investigated. For vertical compressive strains, the charge transfer increases due to the decreased coupling between the layers, whereas tensile strains lead to the opposite behavior. For negative electric fields a transition from the type-III to the type-II band alignment is induced. In contrast, by increasing the positive electric fields, a larger overlap between the valence and conduction bands is observed, leading to a larger band-to-band tunneling (BTBT) current. Low-strained heterostructures with various rotation angles between the constituent layers are also considered. We find only small variations in the energies of the VB and CB edges with respect to the Fermi level, for different rotation angles up to 30°. Overall, our simulations offer insights into the fundamental properties of low-dimensional heterostructures and pave the way for their future application in energy-efficient electronic nanodevices.

## 1 Introduction

The downscaling of the transistors has resulted in improved power efficiencies. However, an additional decrease of the transistor size can cause a significant rise in power consumption and heat production. One of the major issues is the so-called Boltzmann’s limit which restricts the subthreshold swing at 60 mV/dec at room temperature and prevents the reduction of the supply voltage as the size of the transistor is reduced. As a solution to this issue, other transistor architectures, e.g., the tunneling transistors, have been proposed.^1−3^

For traditional transistors, electrons are thermally injected over a potential barrier, whereas for tunnel field-effect transistors (TFETs) the conduction mechanism is the band-to-band tunneling current. Two-dimensional heterostructures with van der Waals interactions between the layers are attractive candidates for future TFETs. Their ideal (free of dangling bonds) interfaces can prevent the trap-assisted tunneling current, and their band alignments can be effectively manipulated by applying gate voltages. In addition, due to the weak interaction between the layers, the constraint of lattice matching is not valid, and appealing van der Waals heterostructures (vdWH) composed of various materials can be realized.^4−10^

Transition-metal dichalcogenide (TMD) monolayers are among the most promising 2D materials for next-generation nanoelectronic applications,^11,12^ and their vertical/lateral stacks have been intensively investigated.^13,14^ 2D ZrS_2_ has been successfully synthesized via an electrochemical lithiation process whereas 2D MoTe_2_ has been fabricated through, e.g., the liquid exfoliating technique.^15,16^ At room temperature, the calculated phonon-limited mobilities are as high as ∼2500 and 1200 cm^2^ V^–1^ s^–1^ for 2D MoTe_2_ and ZrS_2_, respectively.^17^

Through density functional theory (DFT) simulations, the structural and electronic properties of MoTe_2_/ZrS_2_ heterostructures with various stacking patterns are explored. Our simulations show that the valence band (VB) edge of MoTe_2_ is almost aligned with the conduction band (CB) edge of ZrS_2_, and the heterostructures exhibit the type-III or broken gap band alignment, which is pivotal for realizing tunneling transistors. The effects of external electric fields and strain on the electronic properties are also investigated.

It is worth noting that contrary to many theoretical studies focusing on hypothetical 2D materials, our work is devoted to 2D materials which are stable and can be experimentally synthesized. Furthermore, many theoretical investigations consider only one model for the van der Waals heterostructure and limit their analysis to single layers whereas experimentalists may have difficulties to reach the monolayer regime. For instance, MoTe_2_/SnSe_2_ heterostructures have been proved to be promising for future tunneling transistors, but the investigation was focused only on monolayer materials.^18^ Our study overcomes these limitations, and our work can inspire experimentalists to realize tunneling transistors with optimal performance.

## 2 Models and Computational Methods

Our calculations were performed using DFT as implemented in the Vienna Ab initio Simulation Package (VASP).^19,20^ We employed the projected augmented wave (PAW) pseudopotentials^21^ with valence electron configurations of 4s^2^4p^6^4d^5^5s^1^ for Mo, 5s^2^5p^4^ for Te, 4s^2^4p^6^4d^2^5s^2^ for Zr, and 3s^2^3p^4^ for S. Low-strained heterobilayers were constructed using the Cellmatch code,^22^ and periodic slabs with ∼20 Å vacuum were considered. Through the rev-vdW-DF2 functional, dispersion corrections were utilized in the optimizations and total energy computations.^23^ The rest of the calculations were performed using the Perdew, Burke, and Ernzerhof (PBE) functional.^24^

The structures were optimized using the conjugate gradient method with 0.01 eV/Å force convergence criteria and 10^–8^ eV energy convergence criteria. The kinetic energy cutoff was set to 500 eV, and the Brillouin zone was sampled by a 4 × 4 × 1 *k*-mesh for the low-strained heterobilayers consisting of 75 atoms, whereas a 16 × 16 × 1 *k*-mesh was considered for the (1 × 1) MoTe_2_/(1 × 1) ZrS_2_ heterostructures. Energy convergence tests with respect to the number of *k*-points have been performed, and we concluded that a 4 × 4 × 1 *k*-grid results in well-converged results. Furthermore, the effects of relativistic interactions on the electronic properties were investigated. We computed the band structures with SOC by employing the PBE generalized gradient approximation and by using the rev-vdW-DF2 relaxed heterostructures, following the methodology in similar studies.^18,25^ To overcome the underestimation of the band gap of standard DFT, hybrid functional calculations were additionally performed using the HSE06 functional.^26^ For calculations under *E*_ext_ and for the calculation of the average electrostatic potential, dipole corrections were also included perpendicular to the *ab*-plane.

## 3 Results and Discussion

### 3.1 Structural and Electronic Properties of (MoTe_2_)_*m*_/(ZrS_2_)_*m*_ (*m* = 1, 2) vdWH

**Figure 1:**
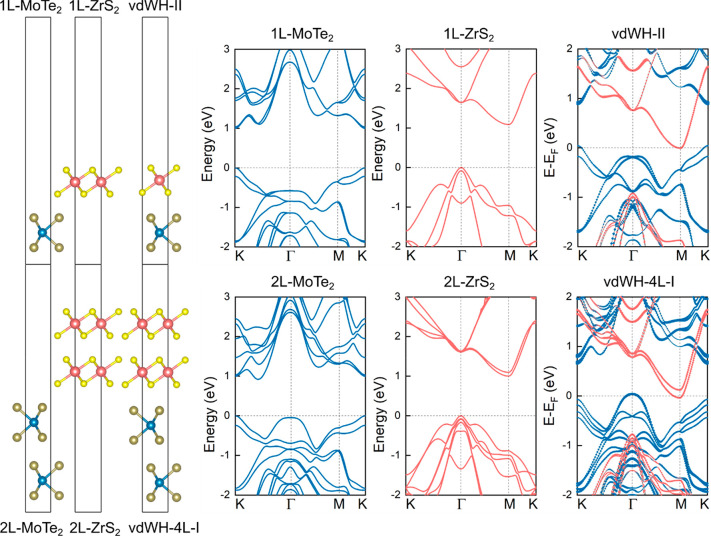
Relaxed atomic structures and electronic band structures of monolayer MoTe_2_ (1L-MoTe_2_), monolayer ZrS_2_ (1L-ZrS_2_), and their favorable heterostack (vdWH-II) (upper panels). Relaxed atomic structures and electronic band structures of bilayer MoTe_2_ (2L-MoTe_2_), bilayer ZrS_2_ (2L-ZrS_2_) and their favorable heterostack (vdWH-4L-I) (lower panels). Blue, brown, orange and yellow spheres correspond to Mo, Te, Zr, and S atoms, respectively. For the band structures of the single-layer and bilayer systems the energies refer to the VB edge. SOC is included in the calculations.

As shown in Figure 1, monolayers MoTe_2_ and ZrS_2_ consist of two hexagonal chalcogen planes separated by one hexagonal metal plane. MoTe_2_ adopts the trigonal-prismatic geometry whereas ZrS_2_ the octahedral one. The in-plane lattice constants are 3.530 and 3.659 Å for MoTe_2_ and ZrS_2_ respectively, and their lattice mismatch is ∼4%. As a first step, we construct heterobilayers by simply stacking MoTe_2_ and ZrS_2_ unit cells and by equally distributing the strain within the layers; i.e., MoTe_2_ is stretched by ∼2% whereas ZrS_2_ is compressed by the same amount. Optimizations are performed to relax the atomic positions whereas the lattice constants are kept fixed. For our study, we consider configurations with different stacking patterns of high symmetry, namely vdWH-I, vdWH-II, vdWH-III, vdWH-IV, vdWH-V, and vdWH-VI (see the Supporting Information, Figure S1). Our calculations reveal that the most stable structure corresponds to vdWH-II for which Zr atoms are located above Mo atoms whereas top-layer S atoms lie above Te atoms. Notably, the total energy of vdWH-IV is only 0.07 meV/atom larger as compared to the lowest energy structure. For vdWH-IV, the bottom-layer S atoms are above Mo atoms whereas the top-layer S atoms are above Te atoms. To verify that our findings do not depend on the selection of the van der Waals functional, OptB86b-vdW and vdW-DF-cx calculations as well as PBE calculations including the Grimme-D3 corrections^27−29^ are also performed, leading to similar results (see the Supporting Information, Table S1).

For the lowest energy structure, the interlayer distance between MoTe_2_ and ZrS_2_ layers is *d*_int_ = 3.1 Å, whereas for all other considered configurations the interlayer distances range from 3.1 to 3.7 Å. These results are also verified by using various van der Waals functionals (see the Supporting Information, Table S1). The calculated interlayer distances are in good agreement with the findings of previous investigations. For instance, Lu et al. performed a systematic study of 120 heterobilayers composed of various TMDs, graphene, and hexagonal boron nitride, and the interlayer distances were found to range from about 3 to 4 Å. In their study, a heterobilayer consisting of 1H-MoTe_2_ and 1H-ZrS_2_ with *d*_int_ = 3.19 Å was also reported.^30^ In our test calculations, considering the same heterobilayer and the same stacking pattern *d*_int_ = 3.10 Å, which is in good agreement with the corresponding value reported in the literature. Notably, the interlayer distances should be predicted as accurately as possible to ensure a proper description of the orbital hybridization, electron charge transfer, electronic structure, etc. A recent paper reports 1H-MoTe_2_/1T-ZrS_2_ heterobilayers having our the so-called vdWH-IV stacking pattern and an interlayer distance of 2.75 Å,^31^ which is slightly lower compared to our computed values using various van der Waals functionals.

Next, the binding energy is calculated through the equation
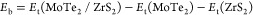
1where *E*_t_(MoTe_2_/ZrS_2_) refers to the total energy of the heterobilayer whereas *E*_t_(MoTe_2_) and *E*_t_(ZrS_2_) refer to the total energies of the isolated (strained) monolayers. For the lowest energy heterobilayer we find *E*_b_ = −23 meV/Å^2^, and the negative sign reveals its structural stability. The binding energies of the other considered configurations are also negative, ranging from −23 to −14 meV/Å^2^.

With regard to the electronic properties, using the PBE functional and including SOC, monolayer MoTe_2_ exhibits a direct band gap of 1.00 eV whereas ZrS_2_ has an indirect band gap of 1.09 eV, in agreement with previously reported simulations.^32^Figure 1 shows the band structure of the lowest energy heterobilayer, and similar results are observed in all other heterobilayers (see the Supporting Information, Figures S2 and S3). The energy difference between the ZrS_2_ conduction band edge and MoTe_2_ valence band edge is *E*_CBM–VBM_ = −0.05 eV. Neglecting spin–orbit coupling the band gaps are found to be slightly larger by 0.13 and 0.04 eV for MoTe_2_ and ZrS_2_, respectively, and for the heterobilayer *E*_CBM–VBM_ is found to be −0.02 eV. DFT typically underestimates the energy gaps, and to overcome this limitation HSE06 calculations are also performed. Using HSE06 and including SOC, the energy difference between the CB and VB edges is −0.02 eV. Overall, our calculations reveal that MoTe_2_/ZrS_2_ heterobilayer exhibits the type-III or broken gap band alignment, with a slight overlap between the VB and CB states, which is highly promising for realizing tunneling transistors.

In line with our findings, Reddy et al. explored heterostructures composed of WSe_2_ and SnSe_2_, and they proved the occurrence of semimetallicity through various transport measurements.^33^ For instance, semimetallicity was revealed by the coexistence of holes and electrons, observed by the Hall slope evolution with lowering the temperature or with sweeping the gate voltage. In their study it was pointed out that the transport properties of WSe_2_/SnSe_2_ interfaces were significantly different compared to the transport properties of the constituent layers which exhibit sizable band gaps. Although our work was focused on heterostructures composed of MoTe_2_ and ZrS_2_, the band alignments of various 2D materials with respect to the vacuum level were also computed (see the Supporting Information, Figure S4), and other combinations of 2D materials that are potentially promising for realizing the type-III band alignment could be identified.

Because of the charge transfer/redistribution and the interfacial dipole formation, the band structure of the heterobilayer is not an exact combination of the band structures of the constituent layers. To accurately assess the effect of stacking on the electronic structure, we compute the band structures of the isolated MoTe_2_ and ZrS_2_ monolayers, having the same lattice parameters as those in the heterobilayer. Considering the heterobilayer, the band gaps of MoTe_2_ and ZrS_2_ are found to be 0.85 and 0.91 eV, respectively, whereas for the noninteracting monolayers the band gaps are 0.86 and 0.87 eV. Furthermore, for the heterobilayer, the MoTe_2_ topmost valence band exhibits a Rashba-like splitting around the Γ-point, which is not observed in the band structure of the isolated system. Although isolated monolayer MoTe_2_ exhibits an out-of-plane mirror symmetry, by placing ZrS_2_ on top of MoTe_2_, the symmetry breaks and the Rashba effect occurs. Considering the KΓ direction, the energy difference and the (maximum) momentum offset between the band extremum around the Γ-point and the band degenerate Γ-point are *E*_R_ = 0.015 eV and *k*_R_ = 0.12 Å^–1^, respectively.

To comment on the transport properties, we calculated the electron effective masses by fitting a parabola to the conduction band minimum.^34^ In particular, the effective masses are derived from the band structures using the formula , where *E*(*k*) is the *k*-resolved energy of the band and  is the reduced Planck constant. For the parabolic fitting a region of 0.08 Å^–1^ is considered.

First, the electron effective masses of the isolated (strain-free) monolayers are computed. For MoTe_2_, the effective masses along the MK and ΓK directions are *m*_e,MK_ = 0.62*m*_0_ and *m*_e,ΓK_ = 0.54*m*_0_. Accordingly, for ZrS_2_ the effective masses in the ΓM and KM directions are m_e,ΓM_ = 1.99*m*_0_ and *m*_e,KM_ = 0.30*m*_0_, and these results are in good agreement with previously reported theoretical calculations.^17^ For the heterobilayer, the CB is originated from ZrS_2_, and considering the energetically favorable configuration, we find *m*_e,ΓM_ = 1.92*m*_0_ and *m*_e,KM_ = 0.26*m*_0_. Overall, heavy effective masses indicate low mobilities, whereas light effective masses correspond to high mobilities.

Heterostructures with various rotation angles between the constituting layers can be experimentally realized.^35−37^ Therefore, we next perform rotations of ZrS_2_ with respect to MoTe_2_, and we look for stacks with the number of atoms <100 and strain <1%. The selected stack consists of  and  layers with a relative rotation between the layers of ∼16°. In this model, MoTe_2_ lattice constants remain unchanged whereas ZrS_2_ accommodates the total strain of ∼0.4%. Such small strain has a minor impact on the electronic structure of single-layer ZrS_2_, and a minor impact on the electronic structure of the heterobilayer is expected. For the selected stack various translation operations have been performed. We move monolayer ZrS_2_ in the *a*/*b* directions, and we consider shifts of 0.2*a* or/and 0.2*b*. Optimizations are performed for every shift, and for the most stable and least stable structures the energy difference is 0.01 meV/atom. The binding energy of the heterobilayer with rotated layers is found to be −18 meV/Å^2^, and the negative sign reveals its structural stability.

**Figure 2:**
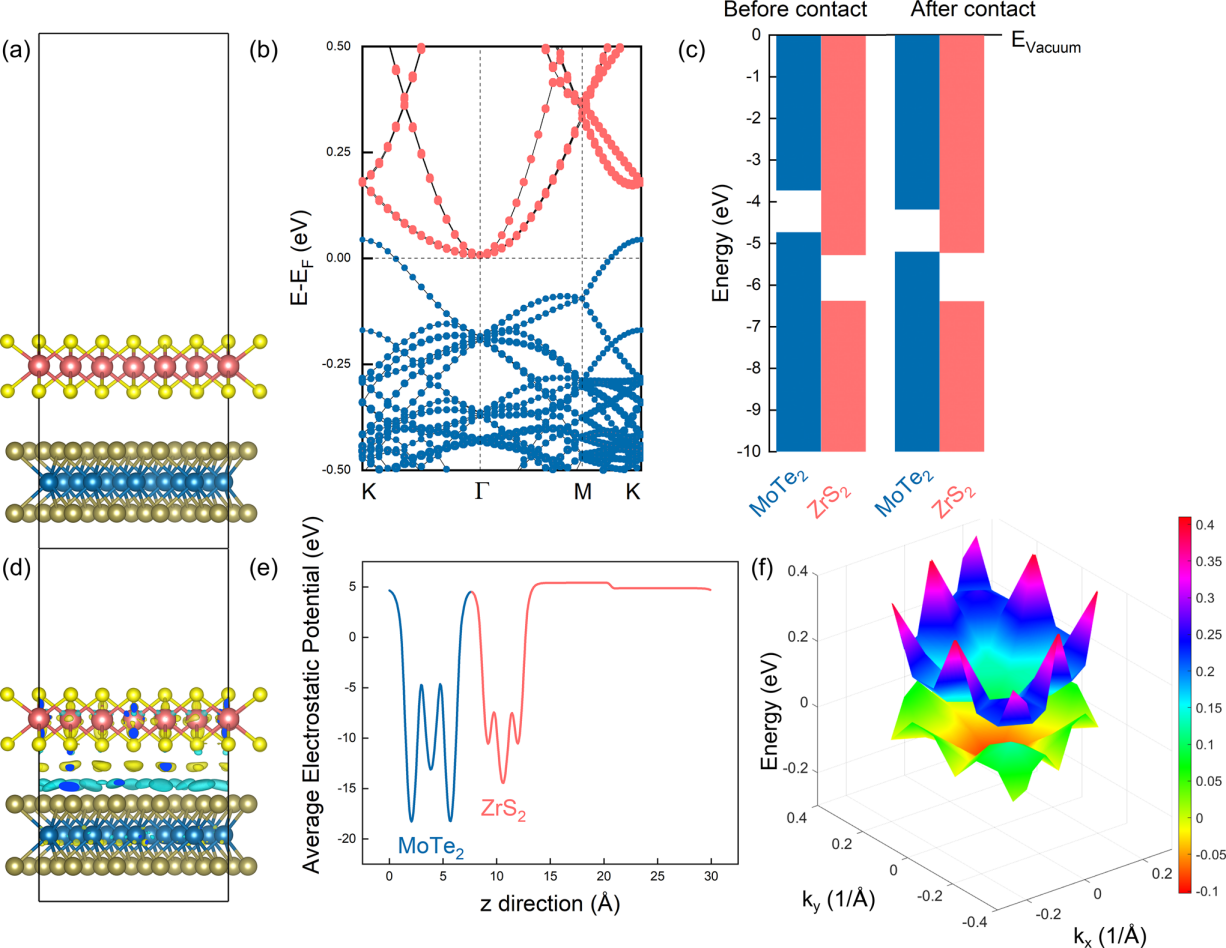
Relaxed atomic structure of low-strained MoTe_2_/ZrS_2_ heterobilayer with rotated layers and its corresponding band structure (a, b). Band alignment with respect to the vacuum level prior and after the heterobilayer formation (c). Charge density difference Δρ_c_ = ρ_c_(MoTe_2_/ZrS_2_) – ρ_c_(MoTe_2_) – ρ_c_(ZrS_2_) and average electrostatic potential along the *z*-axis (d, e). 3D band structure, obtained using VASPKIT,^44^ of the topmost VB and the bottommost CB (f). Blue (orange) lines refer to contributions from MoTe_2_ (ZrS_2_) monolayers. Yellow (blue) isosurfaces correspond to electron accumulation (depletion). The isosurface value is set to 0.0005 electrons/bohr^3^. SOC is included in the calculations.

Figure 2 presents the band structure of the low-strained MoTe_2_/ZrS_2_ vdWH with rotated layers and the band alignment prior and after forming the heterostructure. It is worth mentioning that the band alignment prior to forming the heterostructure corresponds to the unit cells without strain. Using the PBE functional with SOC, the energy difference between the CB and VB edges is found to be −0.04 eV, and the heterobilayer exhibits the type-III band alignment similar to the (1 × 1) MoTe_2_/(1 × 1) ZrS_2_ stack. Because the work function of MoTe_2_ is lower than that of ZrS_2_,^32^ electrons spontaneously flow from MoTe_2_ to ZrS_2_ when the heterostructure is formed. The Bader charge analysis reveals that ∼3 × 10^13^ e/cm^2^ are transferred from the one layer to the other. As a next step, the charge density difference is computed through the formula Δρ_c_ = ρ_c_(MoTe_2_/ZrS_2_) – ρ_c_(MoTe_2_) – ρ_c_(ZrS_2_), where ρ_c_(MoTe_2_/ZrS_2_) refers to the charge density of the heterobilayer whereas ρ_c_(MoTe_2_) and ρ_c_(ZrS_2_) refer to the charge densities of the monolayers. We find that Te atoms of the upper layer and S atoms of the lower layer mainly participate in the electron transfer process. In agreement with the Bader charge analysis, MoTe_2_ and ZrS_2_ exhibit electron depletion and accumulation, respectively, and the heterobilayer resembles an ultrascaled parallel plate capacitor with an intrinsic electric field pointed from MoTe_2_ to ZrS_2_.

Next, heterobilayers with other rotation angles between the constituting layers are briefly discussed. We construct low-strained heterostructures with relative rotations of 10° and 30°, having 177 and 156 total number of atoms, respectively. Using the PBE functional and including SOC, the energy difference between the CB and VB edges is found to be negative, similar to the previously discussed heterobilayers (see the Supporting Information, Figure S5). Overall, our simulations reveal that heterostructures composed of 2D MoTe_2_ and ZrS_2_ with different stacking patterns present band alignments which are highly promising for tunneling transistors.

As a next step, we explore thicker heterostructures composed of MoTe_2_ and ZrS_2_ bilayers. As shown in Figure 1, for MoTe_2_ we consider the 2H-stacking where Mo atoms of the top layer are located above Te atoms of the bottom layer, and vice versa. For ZrS_2_ the two layers are placed on top of each other without any shift or rotation, and these stackings correspond to the lowest energy structures.^38−40^ With regard to the structural properties, the in-plane lattice constants of MoTe_2_ and ZrS_2_ bilayers are 3.533 and 3.655 Å, respectively, and their lattice mismatch is ∼3%. Because of the relatively low lattice mismatch, we can construct heterostructures by simply stacking the corresponding unit cells and by equally distributing the total strain within the layers. Similar to the previous analysis, we consider six high symmetry systems, namely vdWH-4L-I, vdWH-4L-II, vdWH-4L-III, vdWH-4L-IV, vdWH-4L-V, and vdWH-4L-VI (see the Supporting Information, Figure S6). We find that the energetically favorable structure corresponds to vdWH-4L-I, for which the bottom layer S atoms are located above Mo atoms whereas the top-layer S atoms lie above Te atoms. Notably, the total energy of vdWH-4L-V is only 0.14 meV/atom larger as compared to the energy of vdWH-4L-I (see the Supporting Information, Table S2). For the most stable structure the interlayer distance is 3.1 Å, whereas for all other systems it ranges from 3.1 to 3.7 Å (see the Supporting Information, Table S2). Also, for the most stable structure the binding energy is −24 meV/Å^2^, which is nearly equal to the binding energy of the favorable heterobilayer.

Our PBE calculations including SOC show that bilayer MoTe_2_ has an indirect gap of 0.92 eV whereas bilayer ZrS_2_ presents an indirect gap of 1.00 eV. For the bilayers the energy gaps are slightly lower as compared to the monolayer systems. Omitting SOC, the band gaps are larger by 0.06 and 0.04 eV for MoTe_2_ and ZrS_2_, respectively. Figure 1 shows the band structure of the energetically favorable heterostructure whereas heterostructures with other stacking patterns present similar band structures (see the Supporting Information, Figures S7 and S8). Interestingly, the heterostructure formed by bilayer MoTe_2_ and bilayer ZrS_2_ exhibits the type-III or broken gap band alignment. This means that in practical realizations of tunneling transistors it may not be crucial to achieve thicknesses in the monolayer regime. Using the PBE functional and taking into account SOC, the energy difference between the ZrS_2_ CB edge and MoTe_2_ VB edge is −0.08 eV, i.e., slightly lower compared to the corresponding values in the studied heterobilayers.

To evaluate the exact effect of stacking on the electronic structure, we compute the band structures of the isolated MoTe_2_ and ZrS_2_ bilayers, having the same lattice parameters as those in the heterostructure. Considering the heterostructure, the band gaps of bilayer MoTe_2_ and ZrS_2_ are found to be 0.62 and 0.74 eV, respectively, whereas for the noninteracting bilayers the gaps are 0.73 and 0.78 eV. It is worth noting that for the strained isolated MoTe_2_ bilayer the VB edge shifts from the K- to the Γ-point, as compared to the strain-free system, and this shift is also observed in the band structure of the heterostructure. Notably, for the heterostructure the two highest energy valence bands (originated from MoTe_2_) present a significant splitting around the K-point which is not observed in the band structure of the isolated MoTe_2_ bilayer or the isolated slightly stained MoTe_2_ bilayer. The splitting of the bands can be attributed to the break of the symmetry upon stacking. For our investigation, we consider the 2H-stacked bilayer having the inversion symmetry which later breaks by the presence of ZrS_2_. Considering the so-called R-stacked bilayer MoTe_2_ which lacks the inversion symmetry, the splitting of the topmost valence bands around the K-point is observed in its band structure (see the Supporting Information, Figure S9).

For the sake of completeness, we explore heterostructures composed of MoTe_2_ monolayers and ZrS_2_ bilayers (or MoTe_2_ bilayers and ZrS_2_ monolayers). For the constructed stacks the strain is equally distributed between MoTe_2_ and ZrS_2_, and various stacking patterns of high symmetry are considered, namely vdWH-3L-I, vdWH-3L-II, vdWH-3L-III, vdWH-3L-IV, vdWH-3L-V, and vdWH-3L-VI (or vdWH-3L′-I, vdWH-3L′-II, vdWH-3L′-III, vdWH-3L′-IV, vdWH-3L′-V, and vdWH-3L′-VI) (see the Supporting Information, Figures S10 and S11). The two energetically favorable configurations correspond to vdWH-3L-II and vdWH-3L-IV (or vdWH-3L′-I and vdWH-3L′-V). These configurations are structurally equivalent to the lowest energy configurations observed by stacking monolayer materials (vdWH-II and vdWH-IV) or bilayer materials (vdWH-4L-I and vdWH-4L-V). The binding energies of the heterostructures are found to be negative, i.e., – 23 meV/Å for vdWH-3L-II and −24 meV/Å for vdWH-3L′-I. Our PBE calculations including SOC reveal that the heterostructures present the type-III band alignment (see the Supporting Information, Figures S12 and S13). The energy difference between the CB and VB edges is −0.06 and −0.07 eV for vdWH-3L-II and vdWH-3L′-I, respectively. For calculations without SOC, we find similar results (see the Supporting Information, Figures S14 and S15).

As a last step, we examine heterostructures composed of MoTe_2_ monolayers and ZrS_2_ trilayers/tetralayers (or ZrS_2_ monolayers and MoTe_2_ trilayers/tetralayers). In line with the previous analysis, the strain is equally distributed between MoTe_2_ and ZrS_2_, whereas only two possible configurations of high symmetry are considered (see the Supporting Information, Figures S16 and S17). These configurations are structurally equivalent to the lowest energy configurations, observed by stacking monolayer materials or bilayer materials or monolayer along with bilayer materials. With regard to the electronic properties, our PBE calculations including SOC show that trilayer and tetralayer MoTe_2_ are semiconductors with indirect band gaps of 0.82 and 0.79 eV, respectively. Accordingly, trilayer and tetralayer ZrS_2_ exhibit indirect band gaps of 0.96 and 0.95 eV, respectively. Notably, the heterostructures present the broken gap band alignment, and for calculations without SOC we find similar results (see the Supporting Information, Figures S18 and S19).

### 3.2 Application of Electric Field

Next, we examine the electronic properties of MoTe_2_/ZrS_2_ heterobilayers in the presence of external electric fields (*E*_ext_). The electric field is imposed in the vertical direction, and its strength varies from −0.4 to 0.4 V/Å. Positive values correspond to electric fields pointed from MoTe_2_ to ZrS_2_, whereas negative values correspond to the opposite orientation. In our study, both optimizations and band structure calculations are performed under the electric field application.

**Figure 3:**
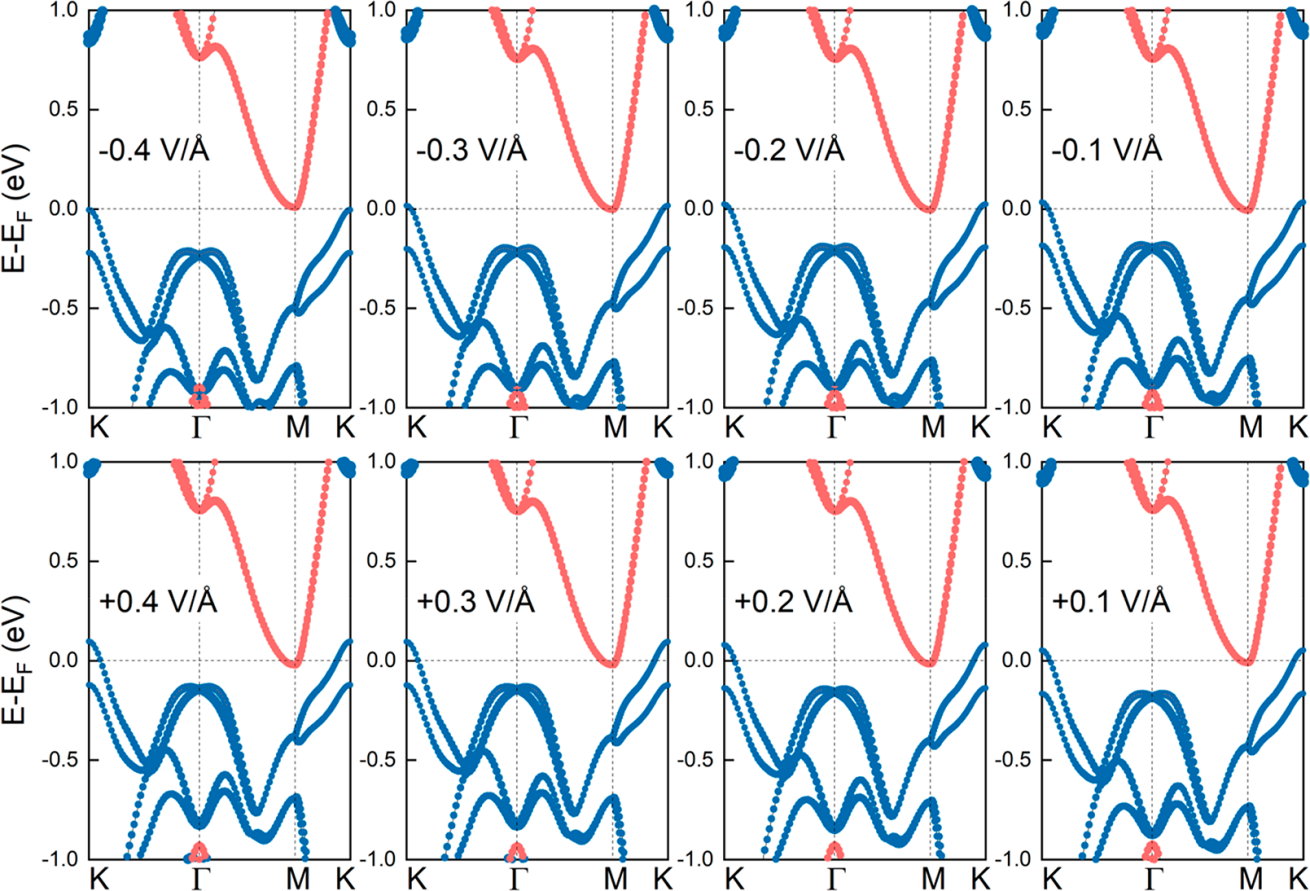
Electronic band structures of (1 × 1) MoTe_2_/(1 × 1) ZrS_2_ heterobilayers in the presence of *E*_ext_. Blue (orange) lines refer to contributions from MoTe_2_ (ZrS_2_) monolayers. SOC is included in the calculations.

The electric field induces a potential gradient normal to the interface and results in the redistribution of the charge carriers along the layers. The charge redistribution can significantly impact the band edge positions, and Figure 3 shows the electronic band structures of (1 × 1) MoTe_2_/(1 × 1) ZrS_2_ heterobilayers under various electric fields. For *E*_ext_ > 0, the band edge positions of MoTe_2_ (ZrS_2_) are pushed upward (downward) with respect to the Fermi level, and by increasing the positive electric fields, the band edge shifts are also increased. Using the PBE functional with SOC, changing the electric field strength from 0.1 to 0.4 V/Å, the energy difference between the ZrS_2_ CB minimum and the MoTe_2_ VB maximum changes from −0.06 to −0.12 eV. Smaller energy differences correspond to larger overlaps between the ZrS_2_ low-lying conduction bands and the MoTe_2_ high-lying valence bands. Consequently, more electrons can tunnel from the one layer to the other. Notably, the opposite behavior is found for electric fields pointed from ZrS_2_ to MoTe_2_, and for negative electric fields a transition from the type-III to the type-II band alignment is observed. Because the interlayer distance of the heterobilayer is ∼3.1 Å, the maximum electric field of 0.4 V/Å corresponds to a potential difference of about 1.2 V. Although this value may appear to be large, it is within the range of electric fields considered in experiments.^41^ For calculations without SOC we find similar results (see the Supporting Information, Figure S20).

**Figure 4:**
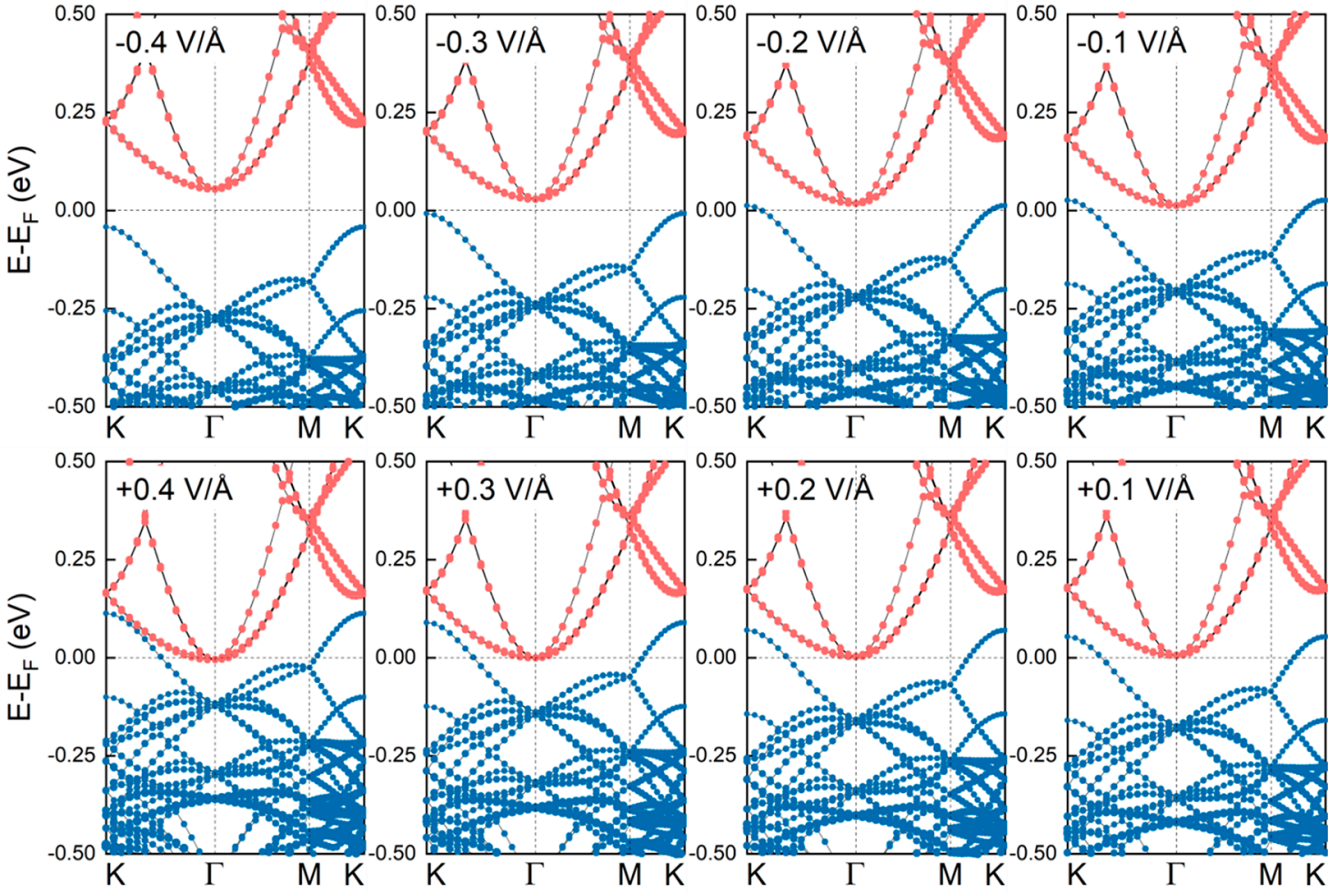
Electronic band structures of low-strained MoTe_2_/ZrS_2_ heterobilayers with rotated layers in the presence of *E*_ext_. Blue (orange) lines refer to contributions from MoTe_2_ (ZrS_2_) monolayers. SOC is included in the calculations.

**Figure 5:**
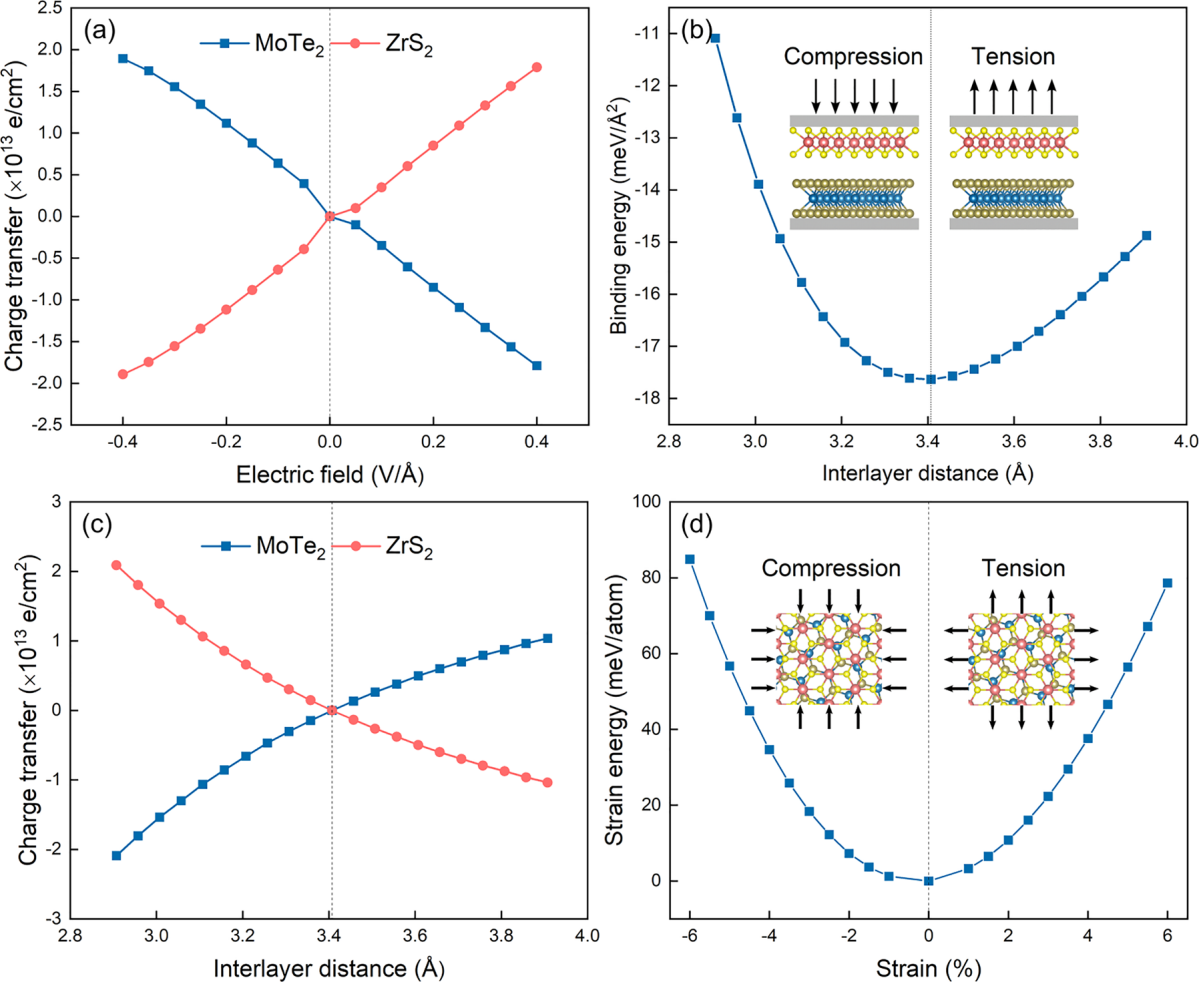
Charge transfers between MoTe_2_ and ZrS_2_ monolayers in the presence of *E*_ext_. The charge of the monolayers in the heterobilayer in the absence of *E*_ext_ is used as a reference (a). Binding energies and charge transfers between MoTe_2_ and ZrS_2_ monolayers under out-of-plane strains. The charge of the monolayers in the strain-free heterobilayer is used as a reference (b, c). Strain energies of heterobilayers under in-plane biaxial strains (d). SOC is included in the calculations.

Similar calculations are also performed for the low-strained heterobilayer with rotated layers. In line with the previous results, positive electric fields lead to the larger overlap between the VB and CB states, whereas negative electric fields result in the opening of the band gap, as shown in Figure 4. For calculations without SOC we find similar results (see the Supporting Information, Figure S21). It is worth mentioning that for this model the interlayer distance is ∼3.4 Å and the maximum electric field of 0.4 V/Å corresponds to a potential difference of ∼1.4 V. Figure 5a shows the Bader charge transfer between the layers in the presence of electric fields of different strengths. For positive (negative) electric fields the electron transfer from MoTe_2_ to ZrS_2_ increases (decreases). These results are consistent with the band structure calculations, and the findings are verified by computing the corresponding charge density differences (see the Supporting Information, Figure S22).

### 3.3 Application of Out-of-Plane and In-Plane Strain

Strain has been widely used for modifying materials properties, and low-dimensional materials can typically tolerate larger strains as compared to their bulk counterparts. For our analysis, low-strain MoTe_2_/ZrS_2_ heterobilayers with rotated layers are considered. As a first step we explore vertical strains, and taking as a reference the ground-state structure, *d*_int_ is either decreased or increased by up to 0.5 Å. Upon relaxation, Te atoms of the upper layer and S atoms of the lower layer can move along the *ab*-plane, and the rest of the atoms can freely move.

Figure 5 shows the binding energies of the strained heterobilayers as well as the Bader charge transfers between the layers. The binding energies of the strained systems are found to be negative, which indicates their structural stability. For tensile strains the electron transfer from MoTe_2_ to ZrS_2_ decreases due to the decreased coupling between the layers, contrary to the results found for compressive strains. Notably, these observations are consistent with the charge density difference computations (see the Supporting Information, Figure S23). With regard to the electronic properties, no major variations are observed in the energies of the VB and CB edges with respect to the Fermi level for the low vertical stain considered in our study. For interlayer distances slightly lower than the equilibrium value of ∼3.4 Å, a small downward shift of the VB edge and a small upward shift of the CB are observed (see the Supporting Information, Figures S24 and S25).

Besides the out-of-plane strain, in-plane strained heterobilayers are also explored. The biaxial in-plane strain is defined by ε = (*a* – *a*_0_)/*a*_0_ × 100%, where *a* and *a*_0_ correspond to the lattice parameters of the strained and pristine heterobilayers, respectively, whereas negative and positive values refer to compression and tension, respectively. Upon relaxation, all atoms are allowed to freely move whereas the lattice parameters remain unchanged.

**Figure 6:**
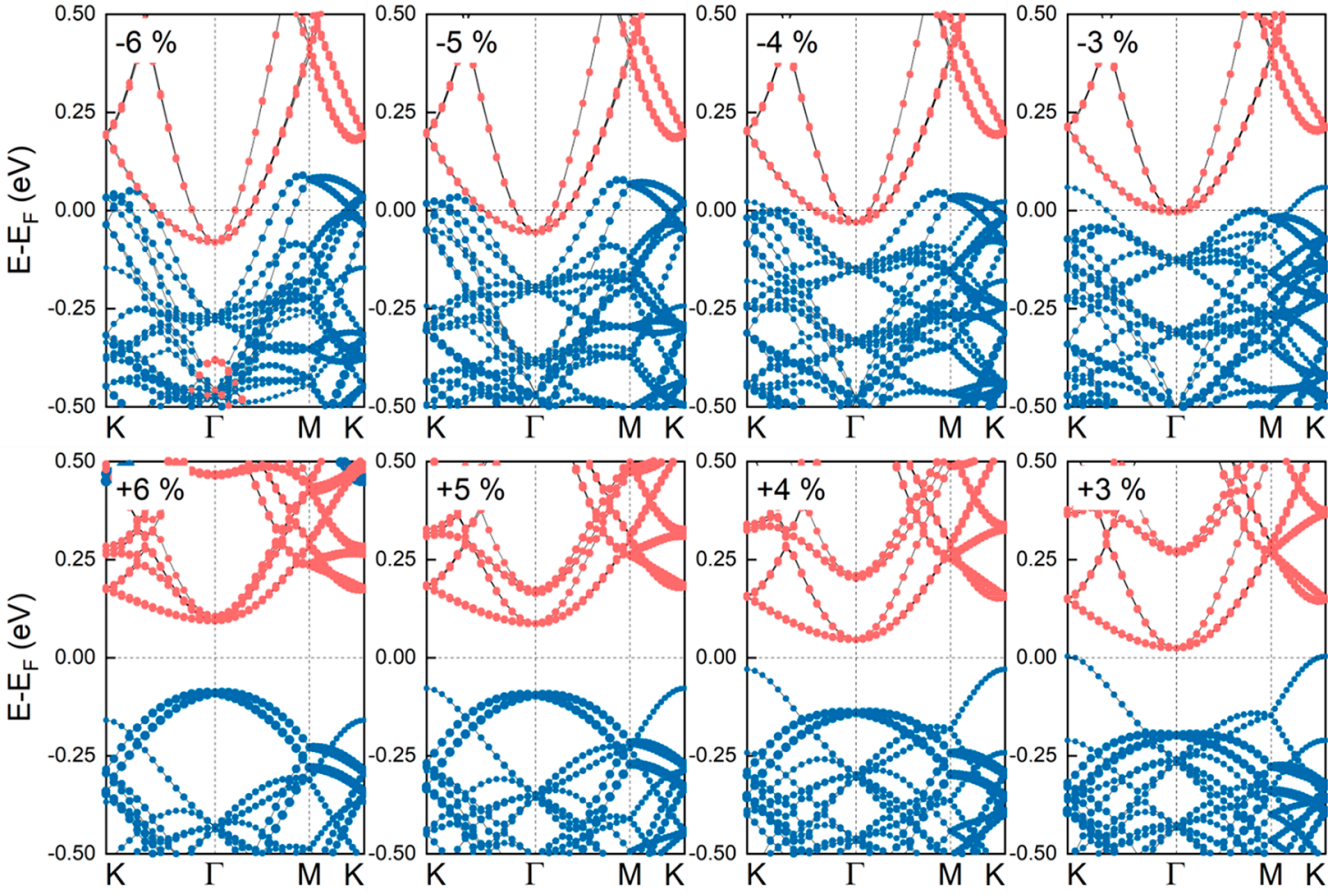
Electronic band structures of low-strained MoTe_2_/ZrS_2_ heterobilayers with rotated layers under in-plane strains. Positive strains refer to tension whereas negative strains refer to compression. Blue (orange) lines refer to contributions from MoTe_2_ (ZrS_2_) monolayers. SOC is included in the calculations.

The strain energies are calculated using the formula *E*_S_ = *E*_st_ – *E*_unst_, where *E*_st_ and *E*_unst_ refer to the total energies of the heterobilayers with and without strain, respectively. As shown in Figure 5d, by increasing the tensile or compressive strains, an increase of the strain energy is observed. Furthermore, *E*_S_ variation resembles a quadratic curve, indicating an elastic deformation of the strained systems. For compressive strains, the high-lying VB states of MoTe_2_ exhibit larger overlap with the low-lying CB states of ZrS_2_, as compared to the strain-free heterobilayer, whereas the opposite behavior is found for tensile strains, as shown in Figure 6. For calculations without SOC we find similar results (see the Supporting Information, Figure S26).

Next, we briefly discuss the impact of strain on the electronic properties of (1 × 1) MoTe_2_/(1 × 1) ZrS_2_ heterobilayers. For the pristine heterobilayer MoTe_2_ and ZrS_2_ are already strained by about 2%, and a stack deformed by, e.g., 4% refers to MoTe_2_ and ZrS_2_ layers deformed by about 6 and 2%, respectively. First, the impact of strain (up to 6%) on the electronic properties of MoTe_2_ and ZrS_2_ isolated layers is investigated. With regard to ZrS_2_, the band gap decreases upon compressive strain and increases upon tensile strain, and these results are in excellent agreement with previously reported theoretical calculations.^42^ Concerning MoTe_2_, tensile strain results in smaller band gaps, and the topmost valence band at Γ-point is shifted upward, which is in line with previously reported findings.^43^ The band structures of the strained heterobilayers are approximate combinations of the band structures of the strained noninteracting monolayers, and the broken gap is preserved for the studied strained systems (see the Supporting Information, Figures S27 and S28).

## 4 Conclusions

In this paper, using first-principles calculations based on density functional theory, we explored the electronic properties of MoTe_2_/ZrS_2_ heterostructures with various stacking patterns and various thicknesses. Our simulations revealed that upon forming the heterostructure electrons spontaneously flow from MoTe_2_ to ZrS_2_, and the system resembles an ultrascaled parallel plate capacitor with an intrinsic electric field pointed from MoTe_2_ to ZrS_2_. We found that the VB and CB edges originated from MoTe_2_ and ZrS_2_ layers, respectively, were almost aligned, leading to the broken gap band alignment. The effects of strain and external electric fields on the electronic properties were also investigated. Notably, by increasing the positive electric fields, a larger overlap between the MoTe_2_ valence bands and the ZrS_2_ conduction bands was observed, leading to a larger band-to-band tunneling current. Low-strained heterostructures with various rotation angles between the constituent layers were also studied, and we found only small variations in the energies of the VB and CB edges with respect to the Fermi level for different rotation angles up to 30°.

## Supporting Information Available

Atomic structures of (1 × 1) MoTe_2_/(1 × 1) ZrS_2_ heterobilayers and their band structures with and without SOC; interlayer distances and relative total energies of (1 × 1) MoTe_2_/(1 × 1) ZrS_2_ heterobilayers; band alignment of various 2D materials with respect to the vacuum level; atomic structures of low-strained heterobilayers with different rotation angles and their band structures with SOC; atomic structures of heterostacks composed of bilayer materials and their band structures with and without SOC; interlayer distances and relative total energies of heterostacks composed of bilayer materials; atomic structure of the R-stacked bilayer MoTe_2_ and its band structure with SOC; atomic structures of heterostacks composed of monolayer and bilayer materials and their band structures with and without SOC; atomic structures of heterostacks composed of monolayer and trilayer/tetralayer materials and their band structures with and without SOC; band structures without SOC of (1 × 1) MoTe_2_/(1 × 1) ZrS_2_ heterobilayers and low-strained heterobilayers in the presence of external electric fields; charge density differences of low- strained heterobilayers under external electric fields and vertical strains; band structures of low-strained heterobilayers under out-of-plane strain with and without SOC; band structures of low-strained heterobilayers under in-plane strain without SOC; band structures of (1 × 1) MoTe_2_/(1 × 1) ZrS_2_ heterobilayers under in-plane strains and the corresponding band structures of the isolated stained monolayers with SOC (PDF)
