# Supplementary material for: Electric Field and Strain Tuning of 2D Semiconductor van der Waals Heterostructures for Tunnel Field-Effect Transistors
Source: ACS Appl Mater Interfaces. 2022 Dec 20;15(1):1762–71. doi: 10.1021/acsami.2c13151 (PMC9837817; doi:10.1021/acsami.2c13151)
Supplement: Supplementary file 1 — am2c13151_si_001.pdf [file am2c13151_si_001.pdf]

Supporting Information

Electric field and strain tuning of 2D semiconductor van der Waals heterostructures for tunnel field-effect transistors

Konstantina Iordanidou[1*], Richa Mitra[2], Naveen Shetty[2], Samuel Lara-Avila[2], Saroj Dash[2], Sergey Kubatkin[2], Julia Wiktor[1]

[1]Department of Physics, Chalmers University of Technology, SE-412 96 Gothenburg, Sweden

[2]Department of Microtechnology and Nanoscience, Chalmers University of Technology, SE-412 96 Gothenburg, Sweden

*Corresponding author: konstantina.iordanidou@chalmers.se

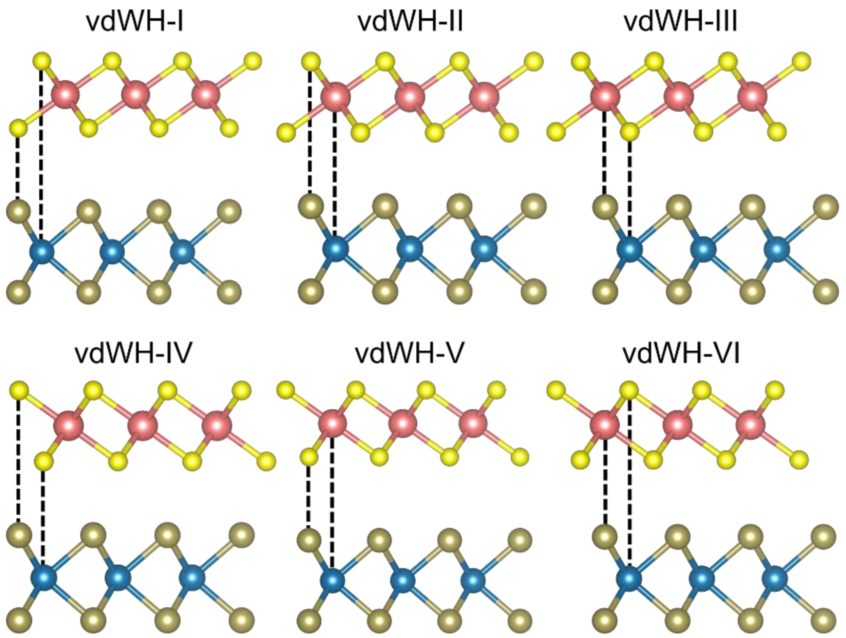

Figure S1: Relaxed atomic structures of $MoTe_2/ZrS_2$ heterobilayers with different stacking patterns of high symmetry.

Table S1: Interlayer distances and relative total energies of $MoTe_2/ZrS_2$ heterobilayers with different stacking patterns. Calculations with various van der Waals functionals and PBE calculations including the Grimme-D3 corrections are presented.

	rev-vdW-D2		OptB86b-vdW		vdW-cx		PBE Grimme-D3	
	d_{int} (Å)	ΔE (meV/atom)	d_{int} (Å)	ΔE (meV/atom)	d_{int} (Å)	ΔE (meV/atom)	d_{int} (Å)	ΔE (meV/atom)
vdWH-I	3.7	15.19	3.7	15.04	3.7	16.41	3.7	13.38
vdWH-II	3.1	0.00	3.1	0.00	3.0	0.00	3.2	0.00
vdWH-III	3.2	3.75	3.2	3.65	3.2	4.27	3.3	3.02
vdWH-IV	3.1	0.07	3.1	0.04	3.1	0.11	3.2	0.06
vdWH-V	3.7	14.44	3.7	14.33	3.7	15.72	3.7	12.70
vdWH-VI	3.3	5.97	3.3	5.74	3.2	6.74	3.3	4.78

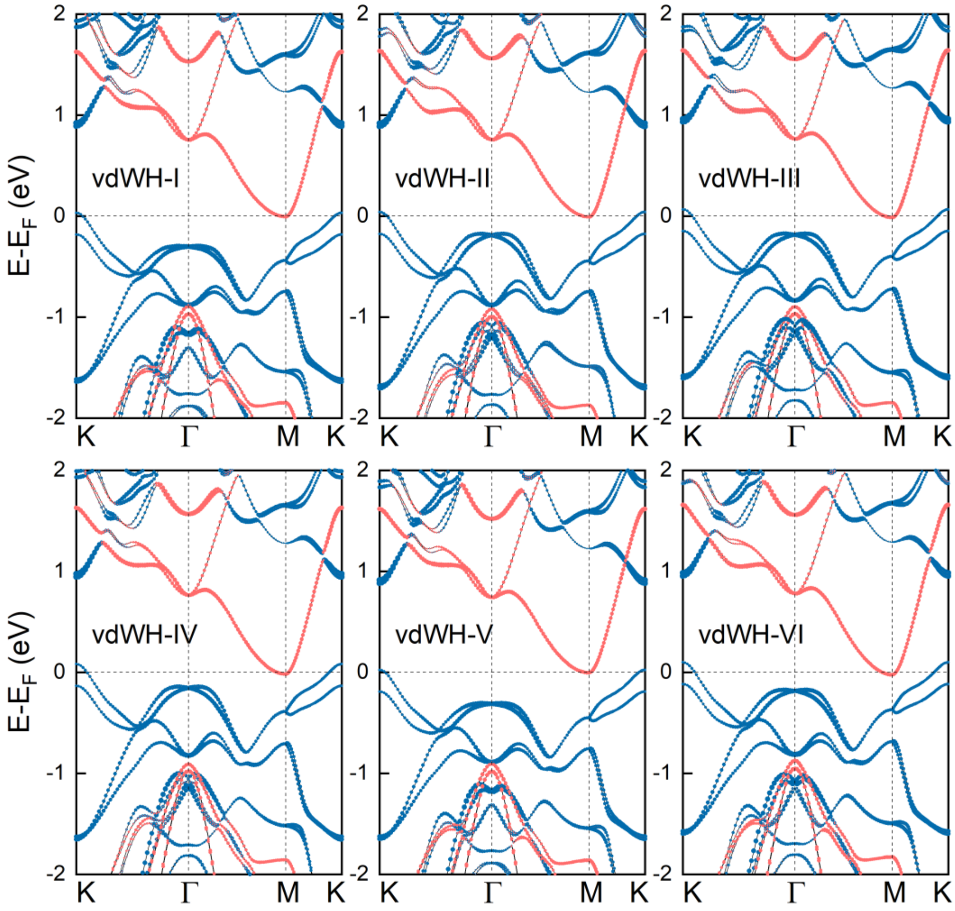

Figure S2: Electronic band structures of $MoTe_2/ZrS_2$ heterobilayers with different stacking patterns. Blue and orange lines refer to contributions from $MoTe_2$ and ZrS_2 layers, respectively. SOC is included in the calculations.

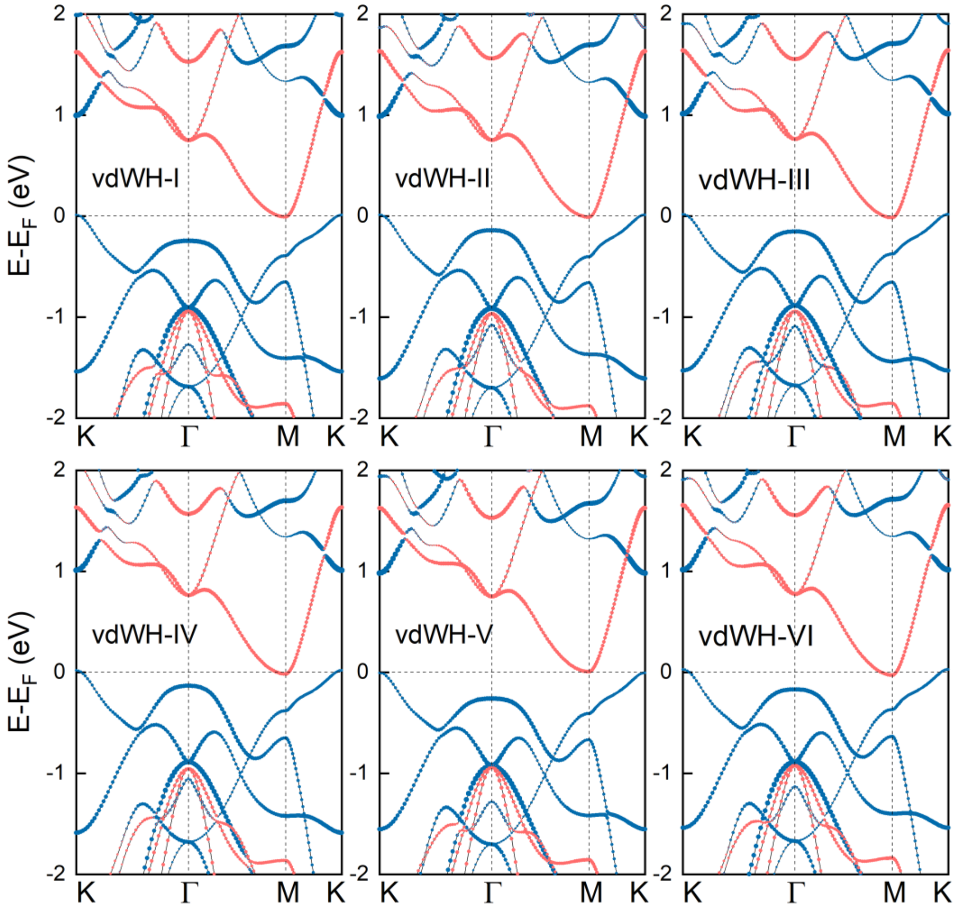

Figure S3: Electronic band structures of $MoTe_2/ZrS_2$ heterobilayers with different stacking patterns. Blue and orange lines refer to contributions from $MoTe_2$ and ZrS_2 layers, respectively. SOC is excluded from the calculations.

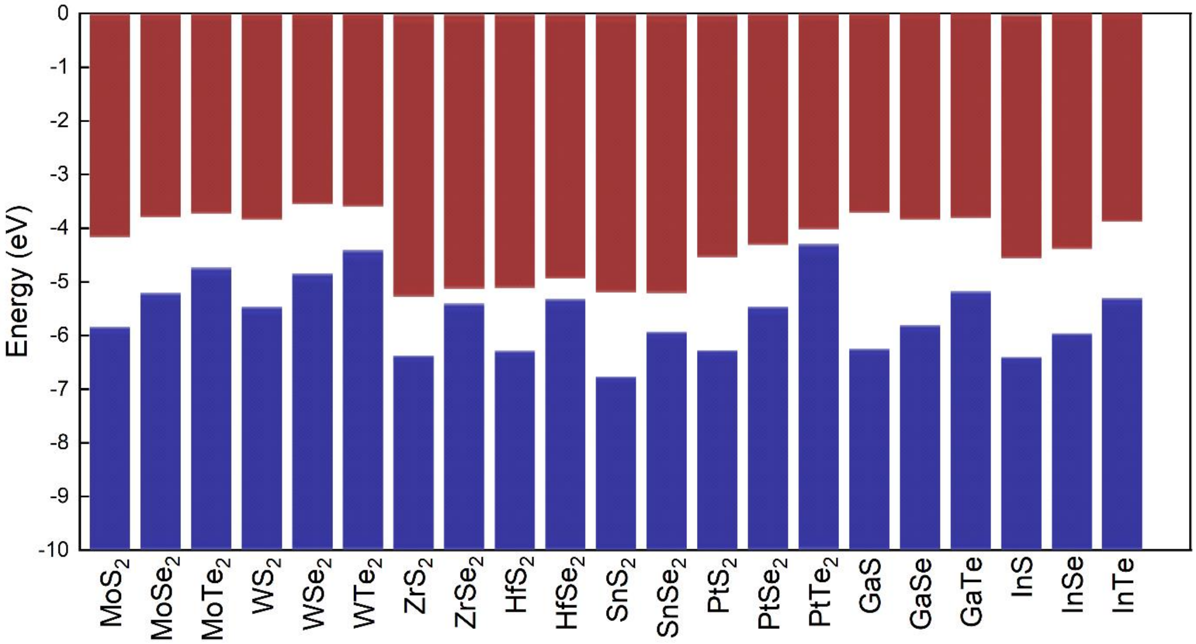

Figure S4: Band alignment with respect to the vacuum level of various two-dimensional materials. SOC is included in the calculations.

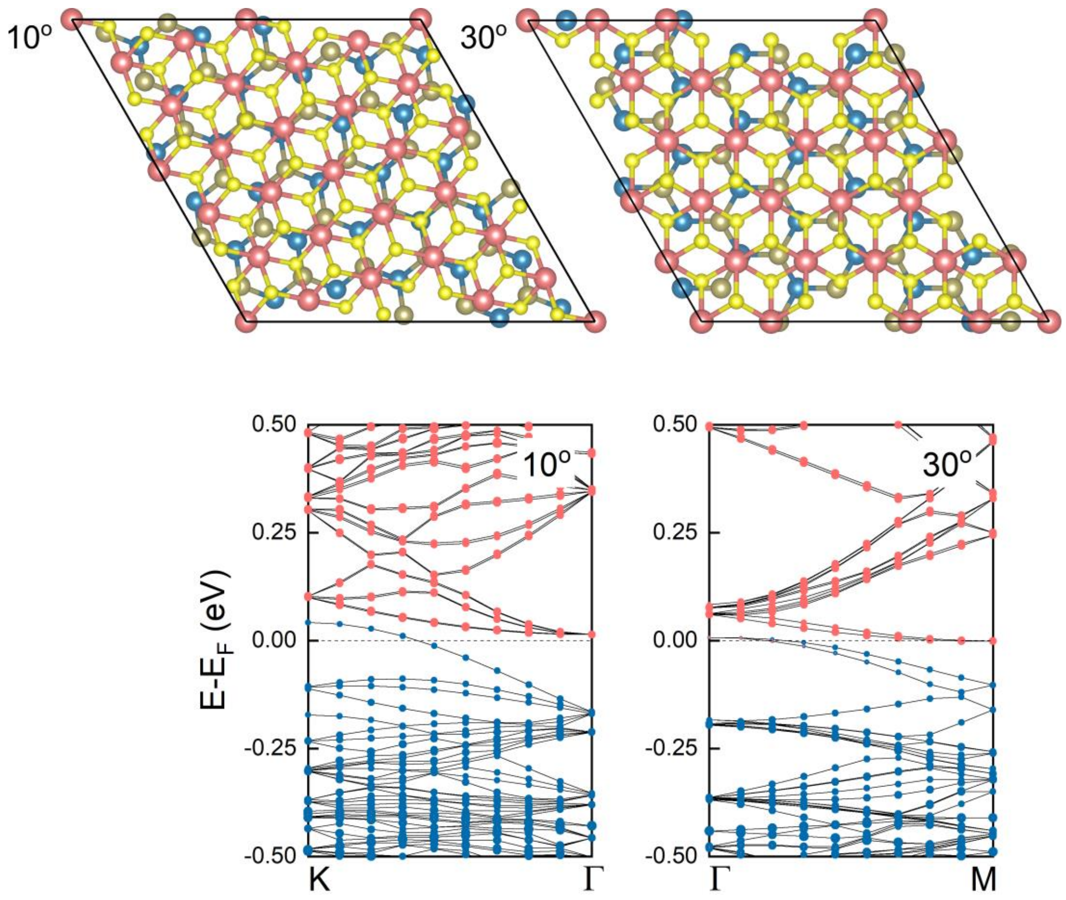

Figure S5: Relaxed atomic structures of $MoTe_2/ZrS_2$ heterobilayers with different rotation angles (upper panels) and their electronic band structures with SOC (lower panels). Blue and orange lines refer to contributions from $MoTe_2$ and ZrS_2 layers, respectively.

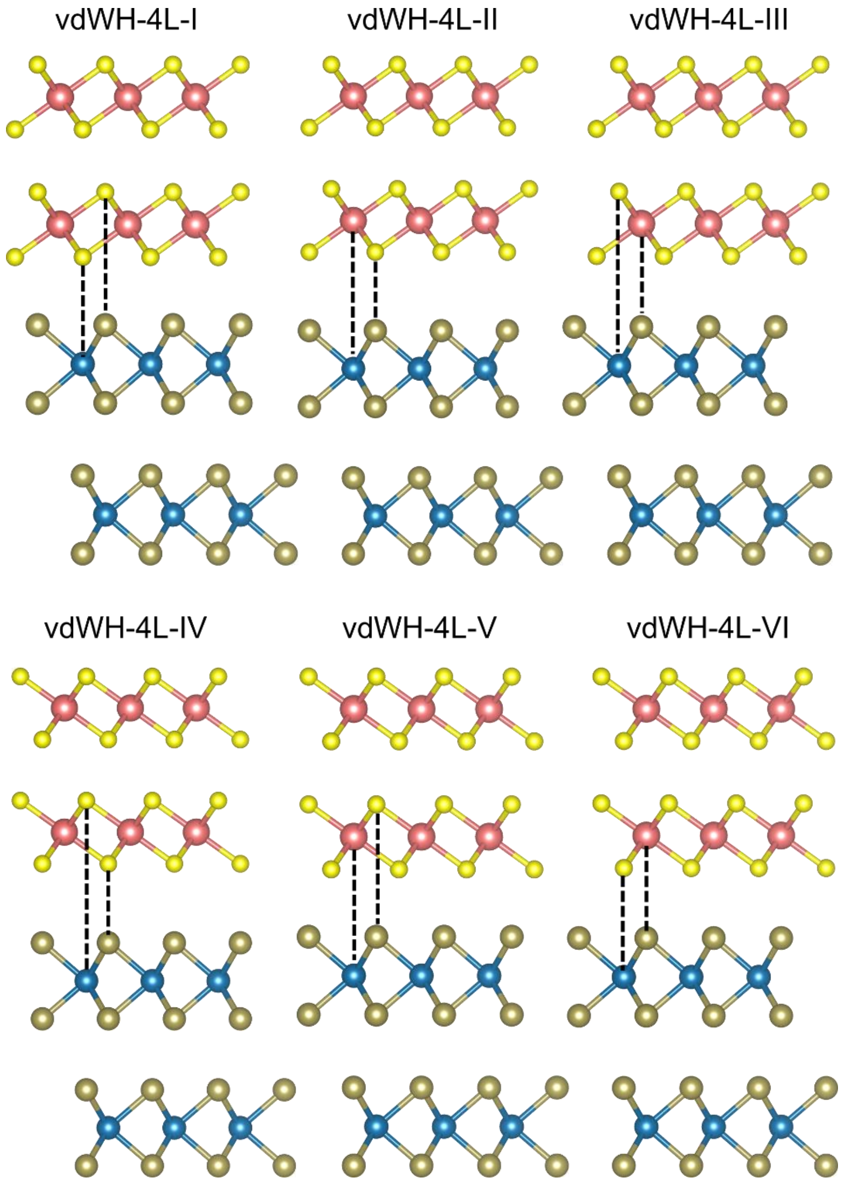

Figure S6: Relaxed atomic structures of heterostacks composed of bilayer $MoTe_2$ and ZrS_2, with different stacking patterns of high symmetry.

Table S2: Interlayer distances and relative total energies of heterostructures composed of bilayer $MoTe_2$ and ZrS_2 with different stacking patterns.

	d_{int} (Å)	ΔE (meV/atom)
vdWH-4L-I	3.1	0.00
vdWH-4L-II	3.7	7.75
vdWH-4L-III	3.3	2.81
vdWH-4L-IV	3.7	7.97
vdWH-4L-V	3.1	0.14
vdWH-4L-VI	3.2	1.84
vdWH-4L-I	3.1	0.00

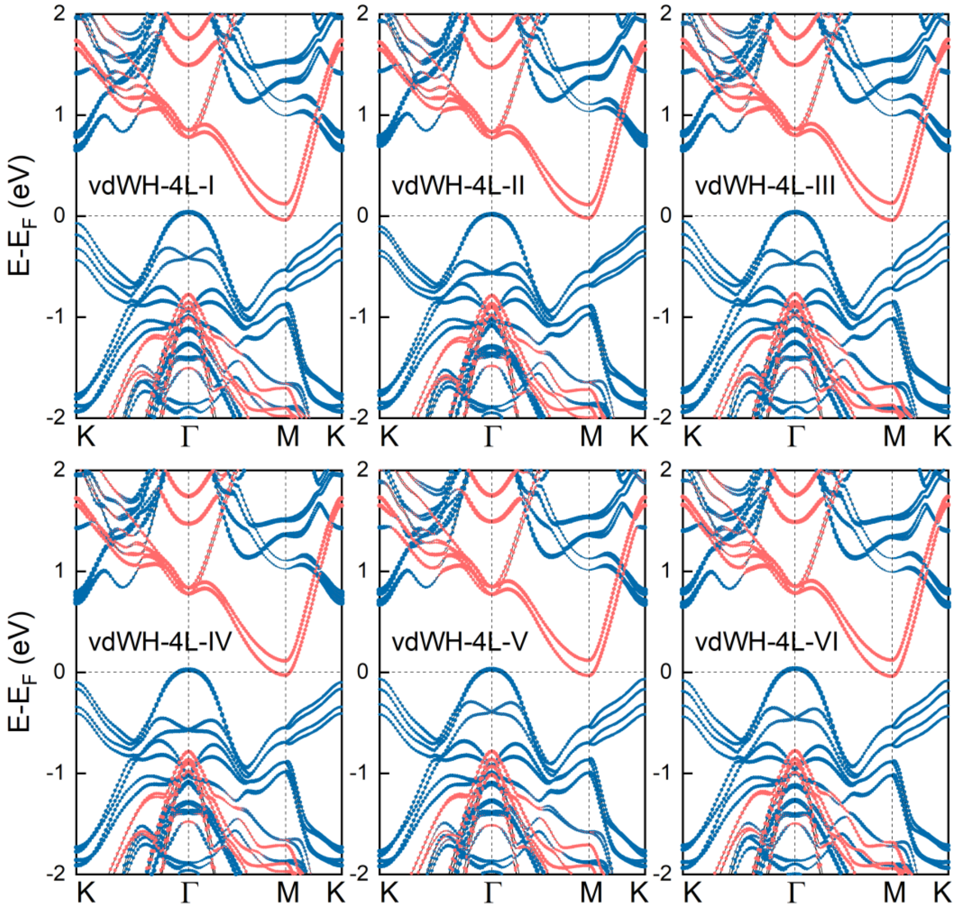

Figure S7: Electronic band structures of heterostructures composed of bilayer $MoTe_2$ and ZrS_2 with different stacking patterns. Blue and orange lines refer to contributions from $MoTe_2$ and ZrS_2 layers, respectively. SOC is included in the calculations.

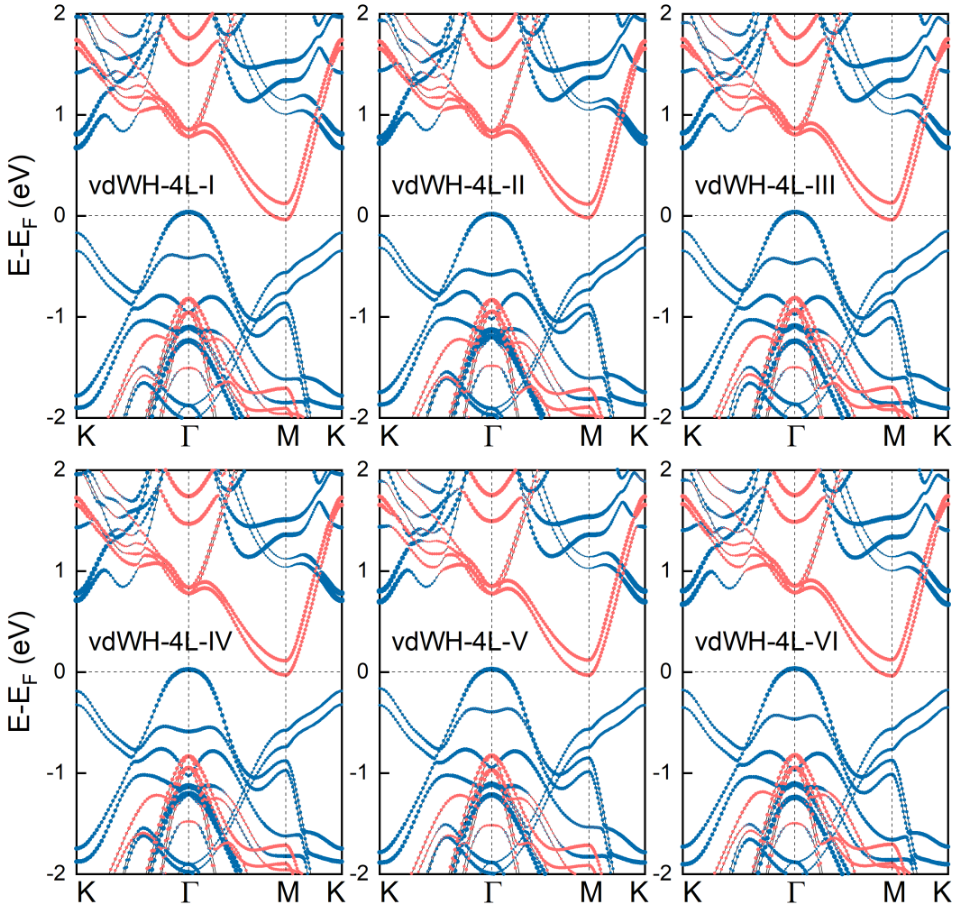

Figure S8: Electronic band structures of heterostructures composed of bilayer $MoTe_2$ and ZrS_2 with different stacking patterns. Blue and orange lines refer to contributions from $MoTe_2$ and ZrS_2 layers, respectively. SOC is excluded from the calculations.

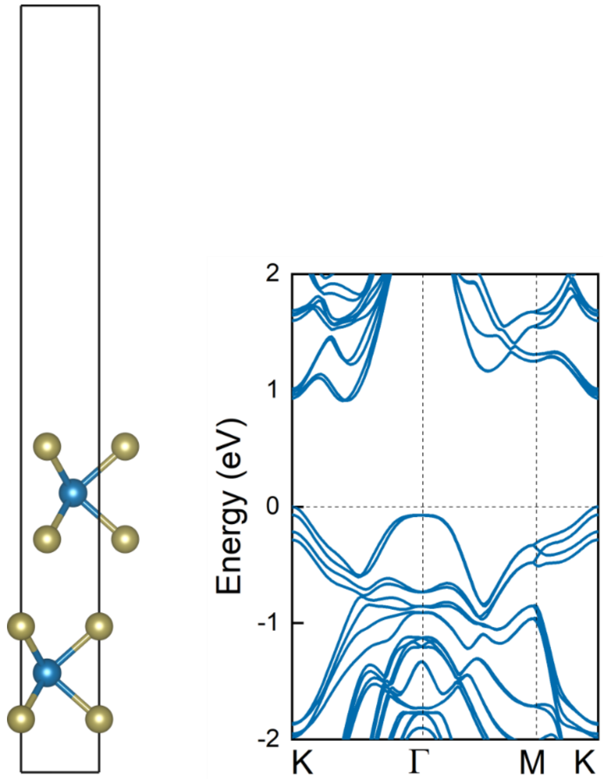

Figure S9: Relaxed atomic structure of the R-stacked bilayer $MoTe_2$ (left panel) and its electronic band structure with SOC (right panel). For the band structure the energies refer to the VB edge.

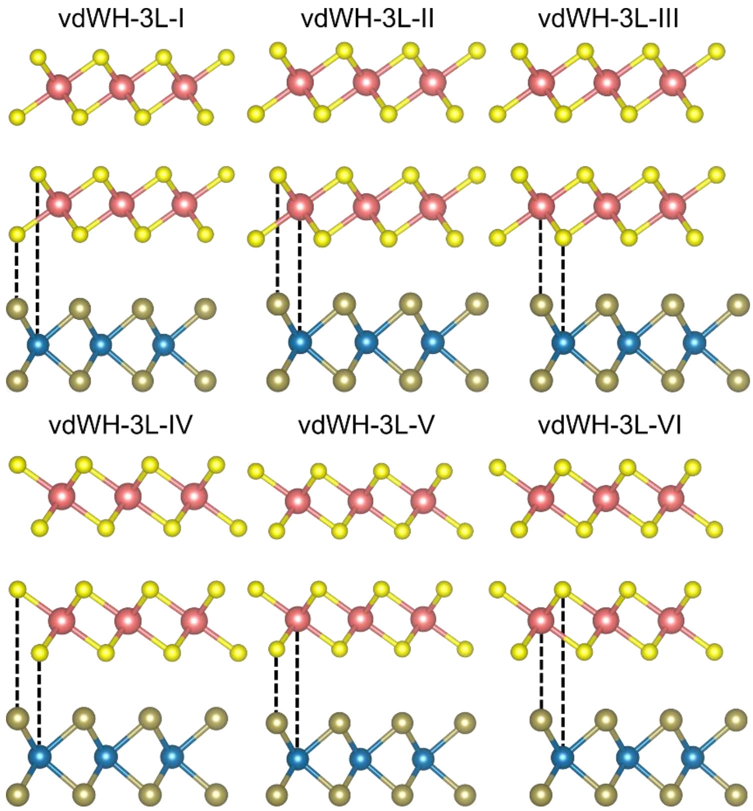

Figure S10: Relaxed atomic structures of heterostacks composed of monolayer $MoTe_2$ and bilayer ZrS_2 with different stacking patterns of high symmetry.

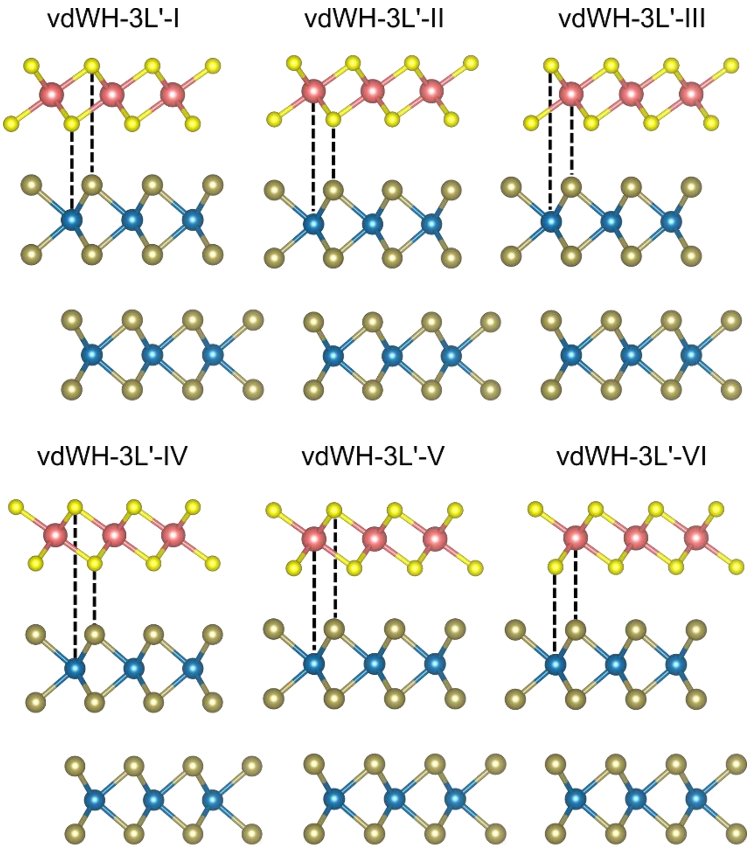

Figure S11: Relaxed atomic structures of heterostacks composed of bilayer $MoTe_2$ and monolayer ZrS_2 with different stacking patterns of high symmetry.

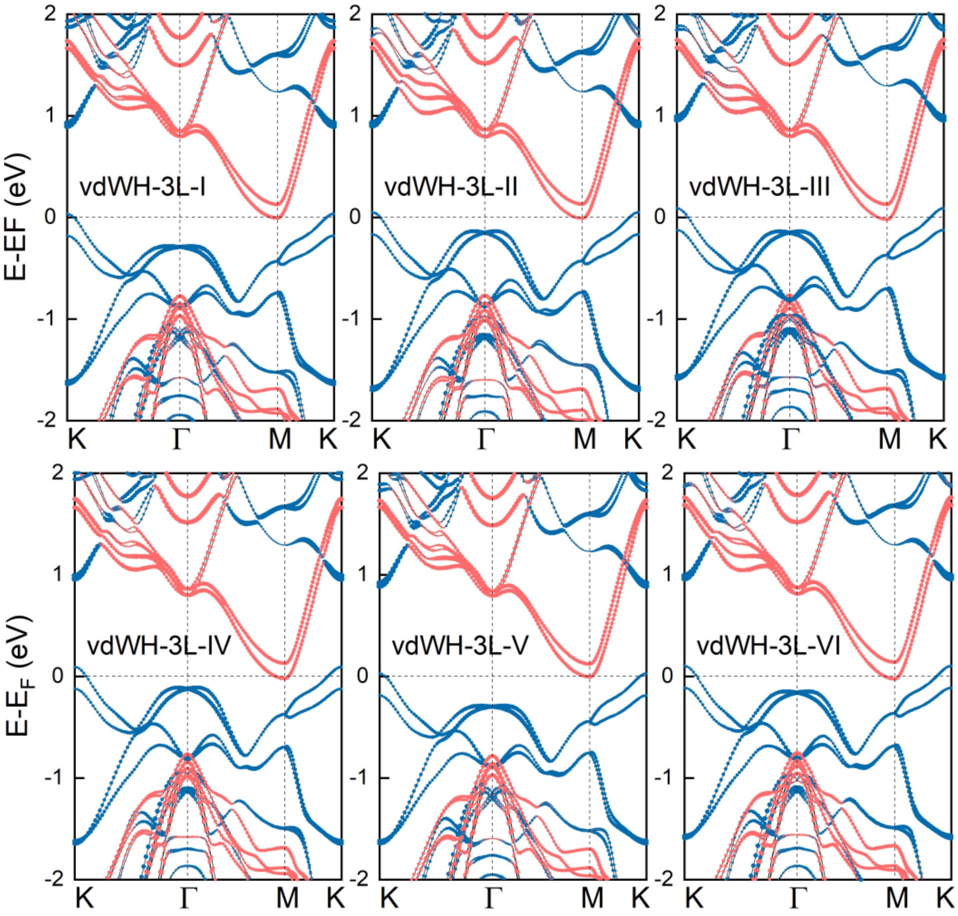

Figure S12: Electronic band structures of heterostructures composed of monolayer $MoTe_2$ and bilayer ZrS_2 with different stacking patterns. Blue and orange lines refer to contributions from $MoTe_2$ and ZrS_2 layers, respectively. SOC is included in the calculations.

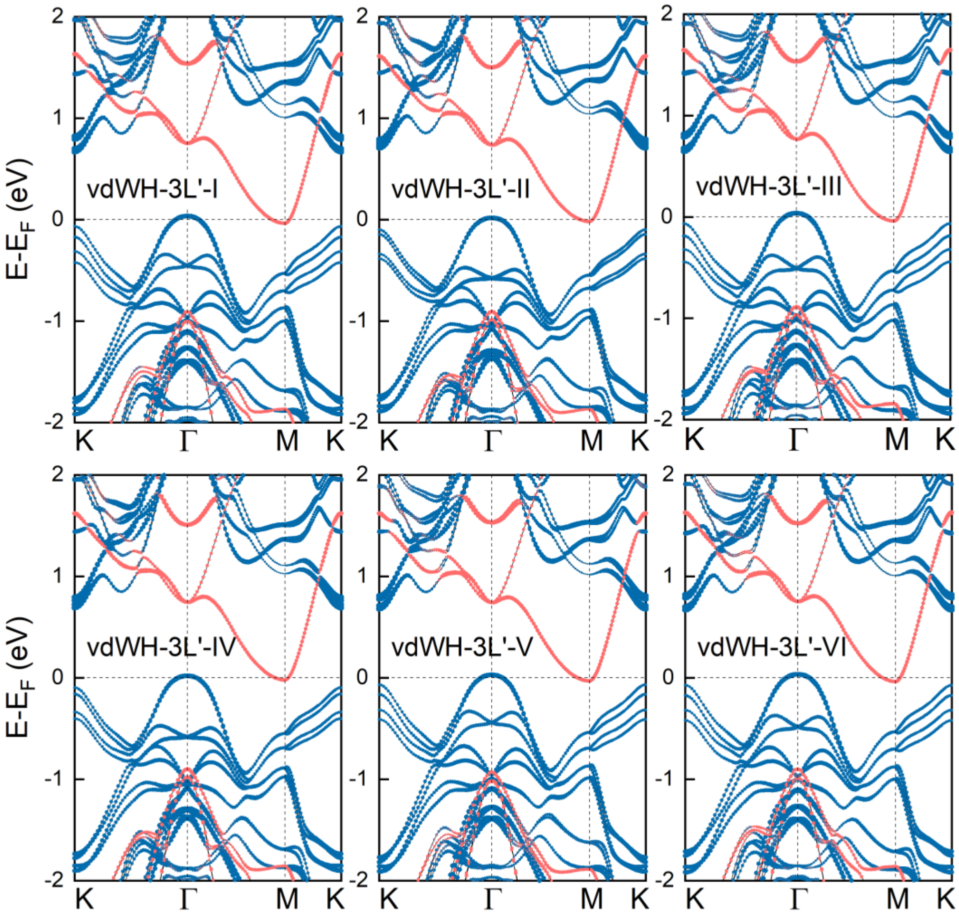

Figure S13: Electronic band structures of heterostructures composed of bilayer $MoTe_2$ and monolayer ZrS_2 with different stacking patterns. Blue and orange lines refer to contributions from $MoTe_2$ and ZrS_2 layers, respectively. SOC is included in the calculations.

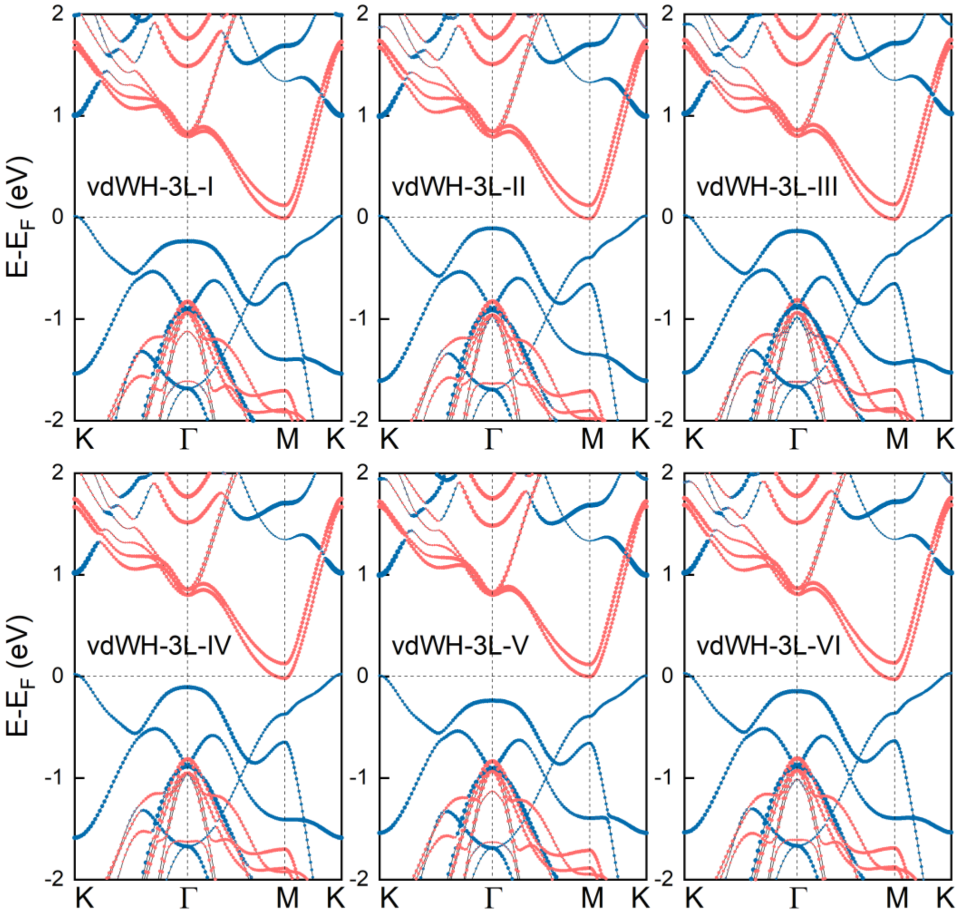

Figure S14: Electronic band structures of heterostructures composed of monolayer $MoTe_2$ and bilayer ZrS_2 with different stacking patterns. Blue and orange lines refer to contributions from $MoTe_2$ and ZrS_2 layers, respectively. SOC is excluded from the calculations.

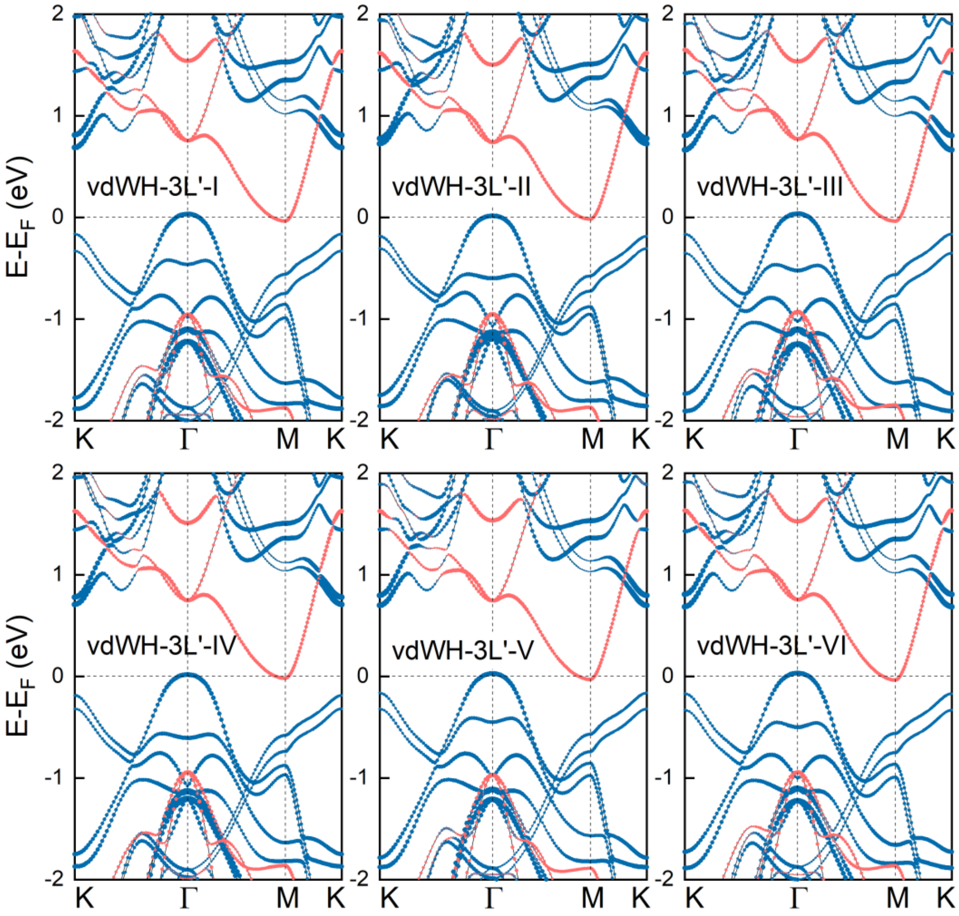

Figure S15: Electronic band structures of heterostructures composed of bilayer $MoTe_2$ and monolayer ZrS_2 with different stacking patterns. Blue and orange lines refer to contributions from $MoTe_2$ and ZrS_2 layers, respectively. SOC is excluded from the calculations.

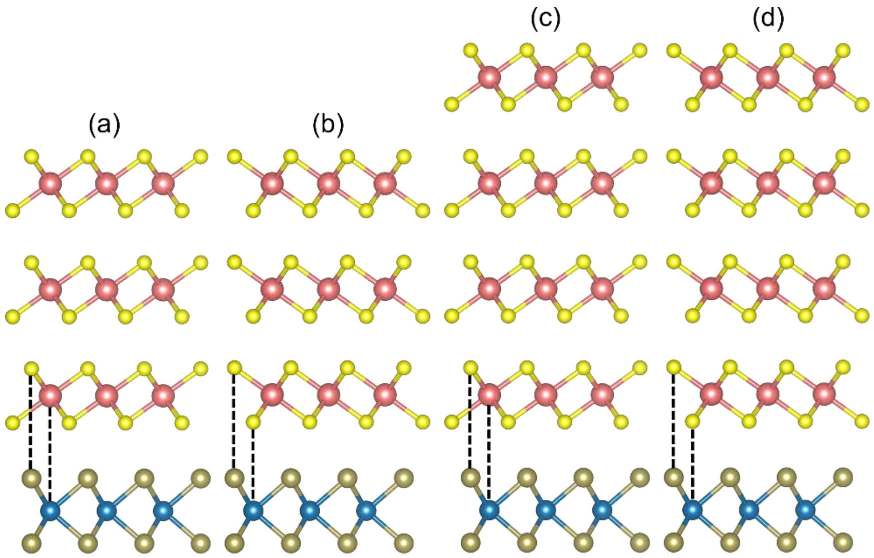

Figure S16: Relaxed atomic structures of heterostacks composed of monolayer $MoTe_2$ and trilayer ZrS_2 (a), (b) as well as monolayer $MoTe_2$ and tetralayer ZrS_2 (c), (d).

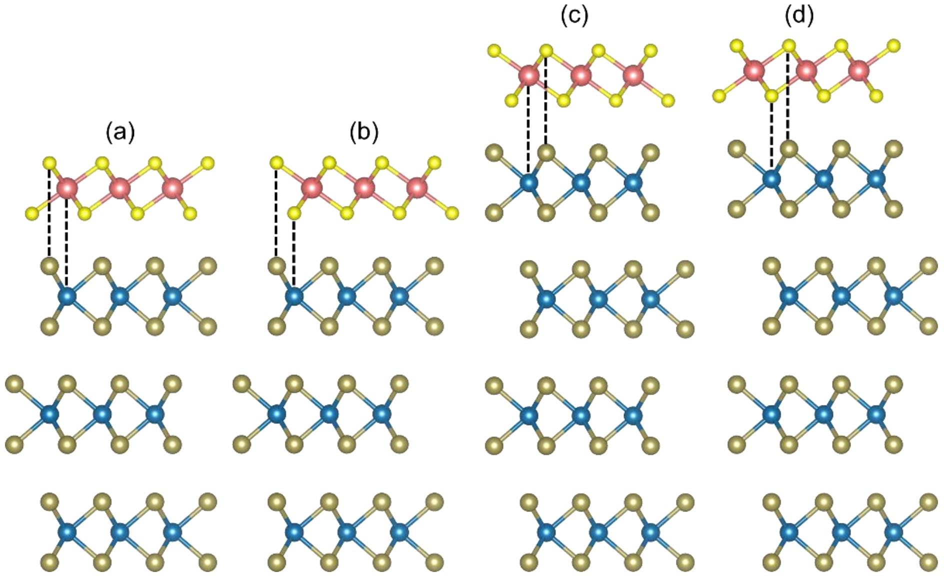

Figure S17: Relaxed atomic structures of heterostacks composed of trilayer $MoTe_2$ and monolayer ZrS_2 (a), (b) as well as tetralayer $MoTe_2$ and monolayer ZrS_2 (c), (d).

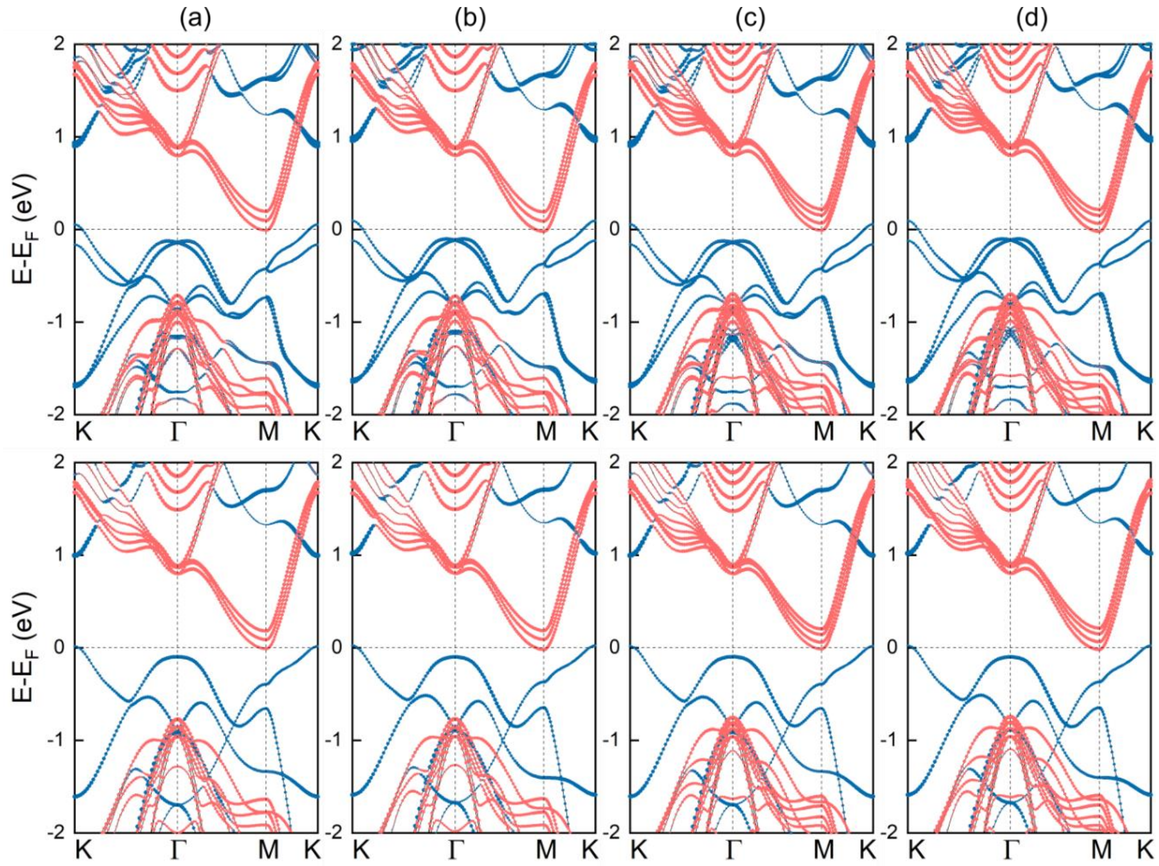

Figure S18: Electronic band structures with SOC (upper panels) and without SOC (lower panels) of heterostructures composed of monolayer $MoTe_2$ and trilayer ZrS_2 (a), (b) as well as monolayer $MoTe_2$ and tetralayer ZrS_2 (c), (d). Blue and orange lines refer to contributions from $MoTe_2$ and ZrS_2 layers, respectively.

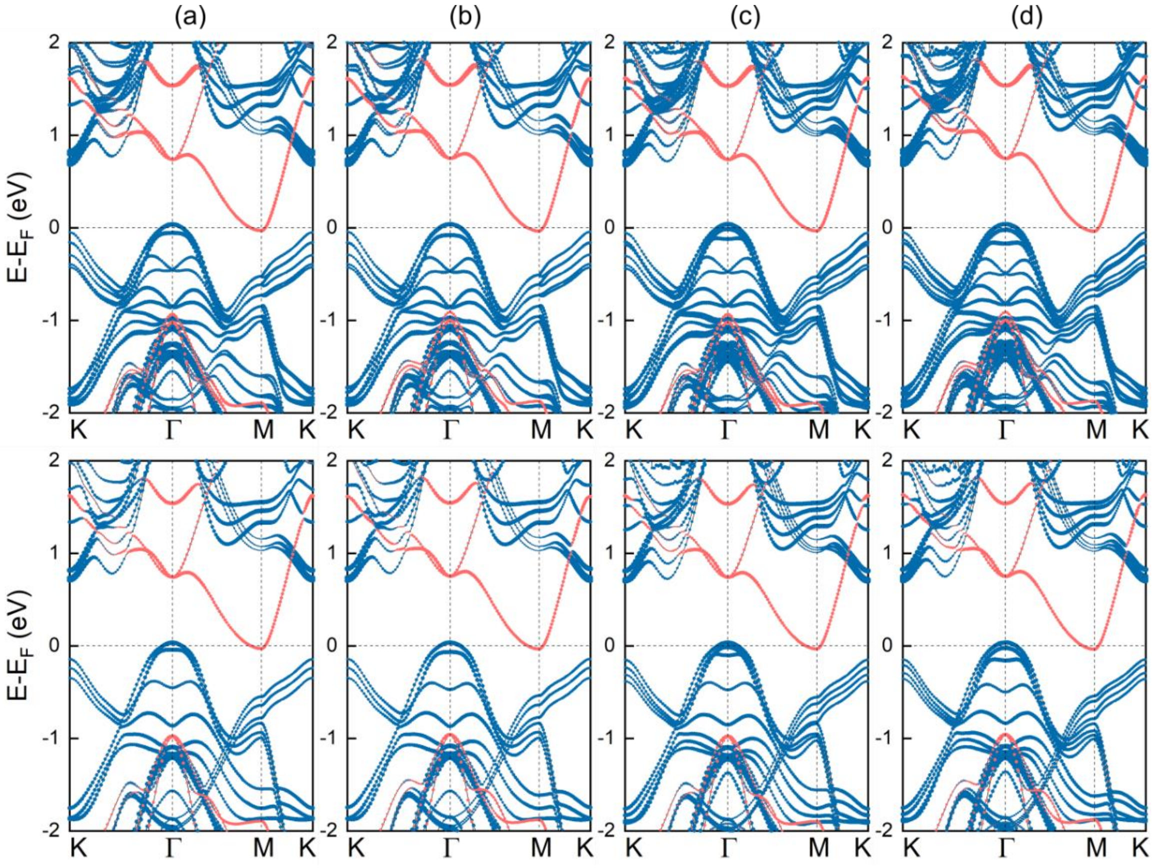

Figure S19: Electronic band structures with SOC (upper panels) and without SOC (lower panels) of heterostructures composed of trilayer $MoTe_2$ and monolayer ZrS_2 (a), (b) as well as tetralayer $MoTe_2$ and monolayer ZrS_2 (c), (d). Blue and orange lines refer to contributions from $MoTe_2$ and ZrS_2 layers, respectively.

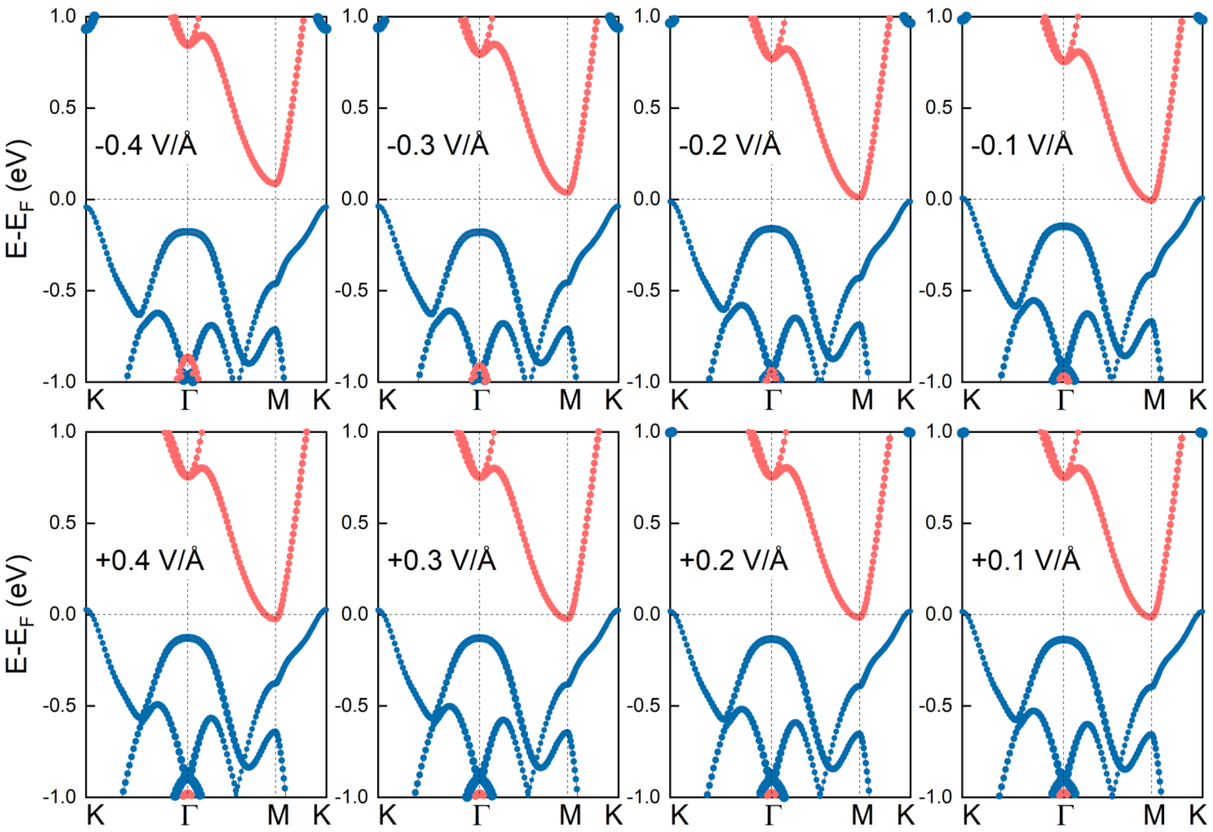

Figure S20: Electronic band structures of (1×1) $MoTe_2$/(1×1) ZrS_2 heterobilayers in the presence of external electric fields. Blue and orange lines refer to contributions from $MoTe_2$ and ZrS_2 layers, respectively. SOC is excluded from the calculations.

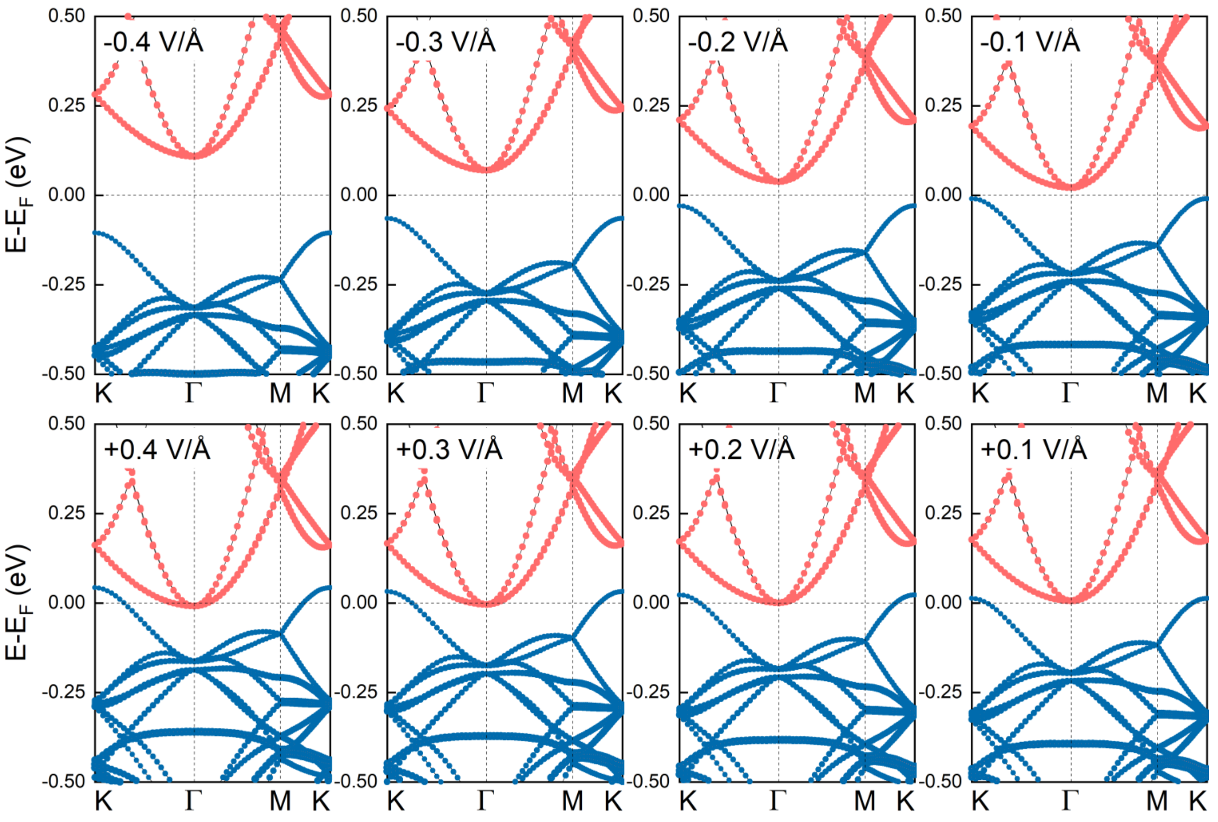

Figure S21: Electronic band structures of low-strained $MoTe_2/ZrS_2$ heterobilayers with rotated layers in the presence of external electric fields. Blue and orange lines refer to contributions from $MoTe_2$ and ZrS_2 layers, respectively. SOC is excluded from the calculations.

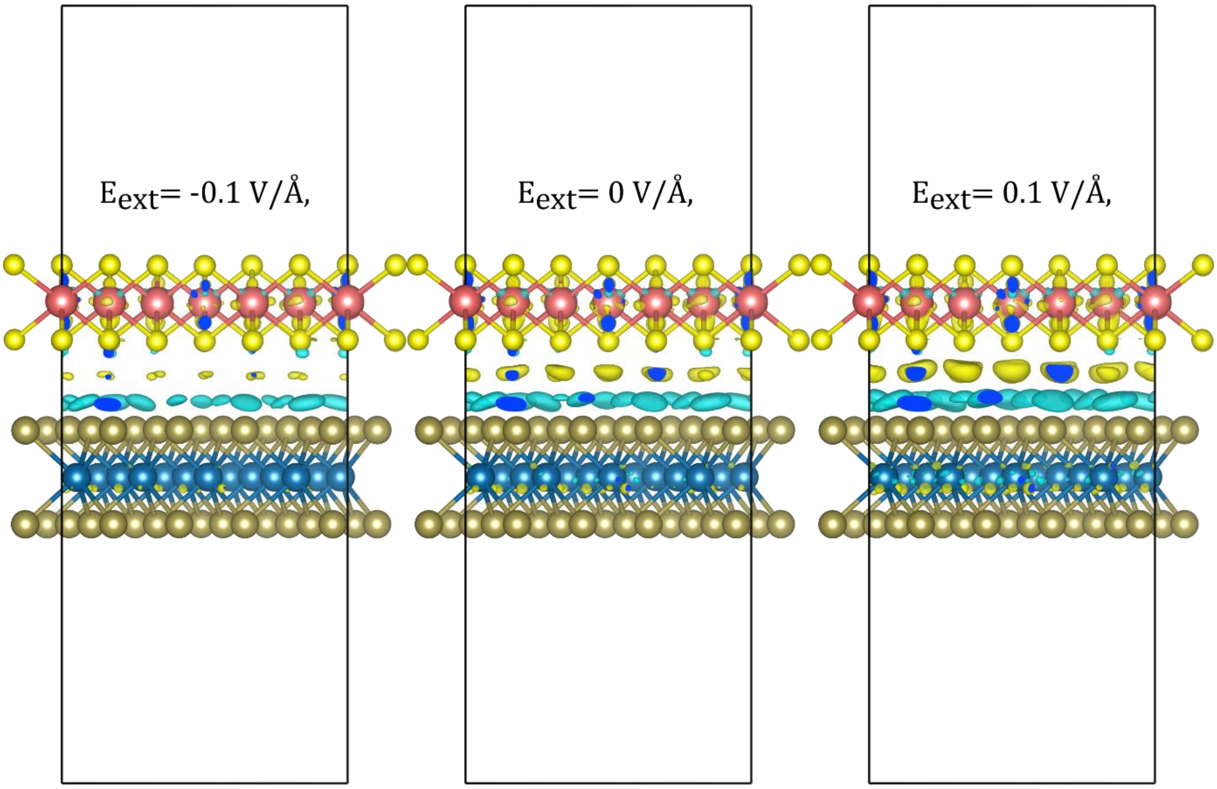

Figure S22: Charge density difference $\Delta\rho_c = \rho(MoTe_2/ZrS_2) - \rho(MoTe_2) - \rho(ZrS_2)$ for different external electric fields. Yellow and blue isosurfaces refer to electron accumulation and depletion, respectively, and the isosurface value is 0.0005 $electrons/Bohr^3$. SOC is included in the calculations.

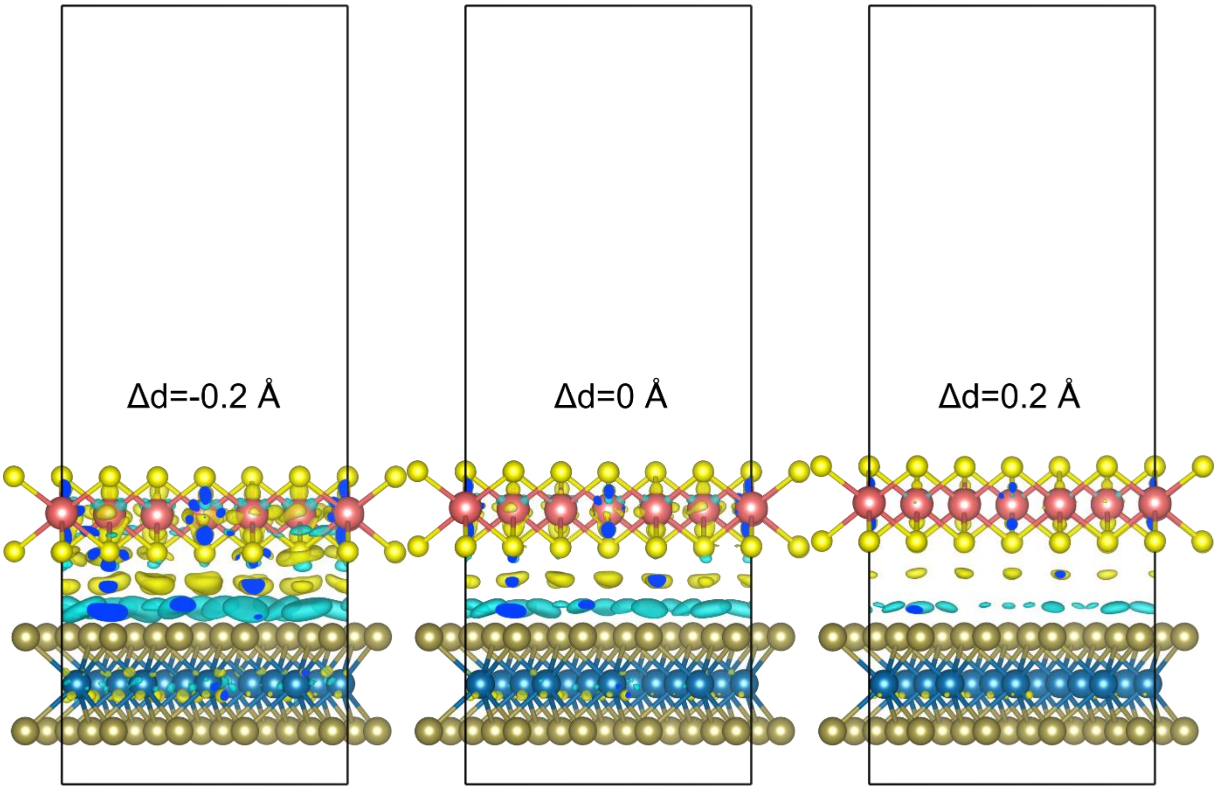

Figure S23: Charge density difference $\Delta\rho_c = \rho(\mathrm{MoTe_2/ZrS_2}) - \rho(\mathrm{MoTe_2}) - \rho(\mathrm{ZrS_2})$ for different vertical strains. Yellow and blue isosurfaces refer to electron accumulation and depletion, respectively, and the isosurface value is 0.0005 electrons/$\mathrm{Bohr^3}$. SOC is included in the calculations.

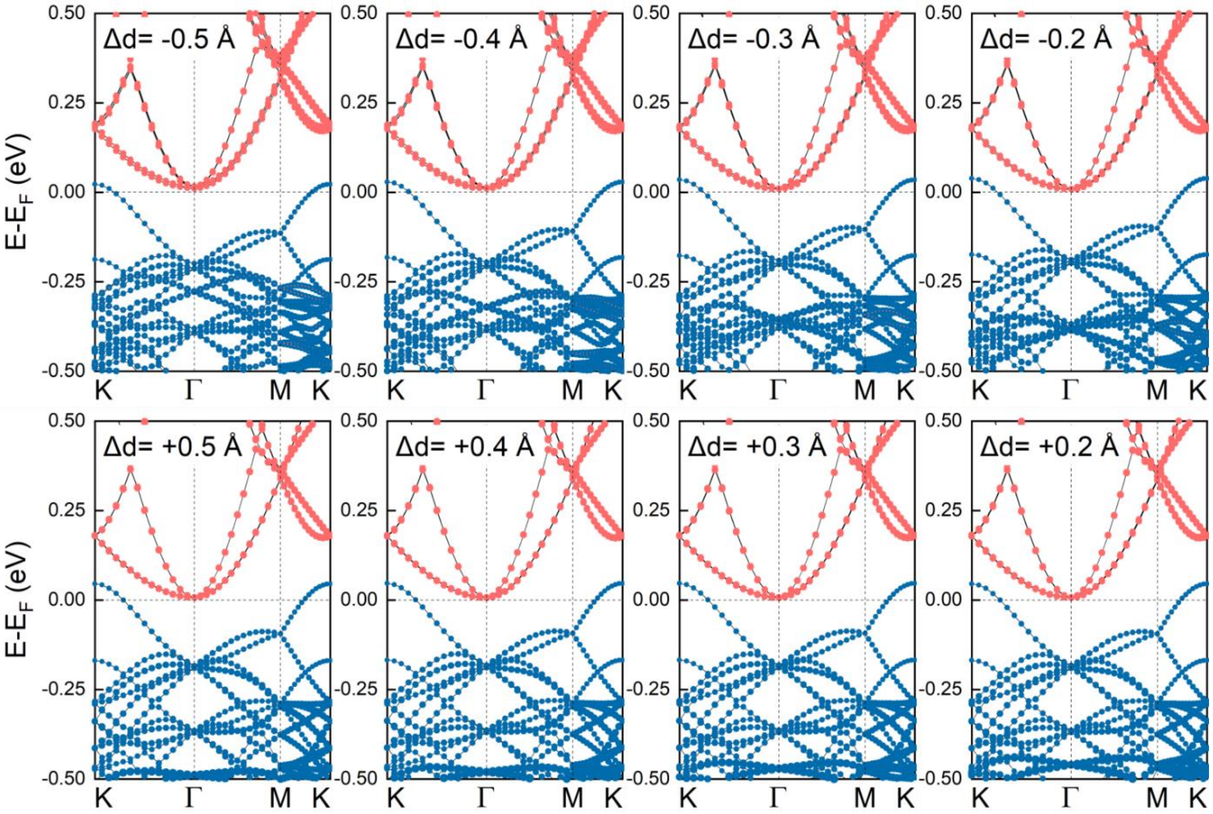

Figure S24: Electronic band structures of low-strained $MoTe_2/ZrS_2$ heterobilayers with rotated layers under out-of-plane strains. Blue and orange lines refer to contributions from $MoTe_2$ and ZrS_2 layers, respectively. Δd corresponds to the interlayer distance variation having as reference the interlayer distance of the ground state structure. SOC is included in the calculations.

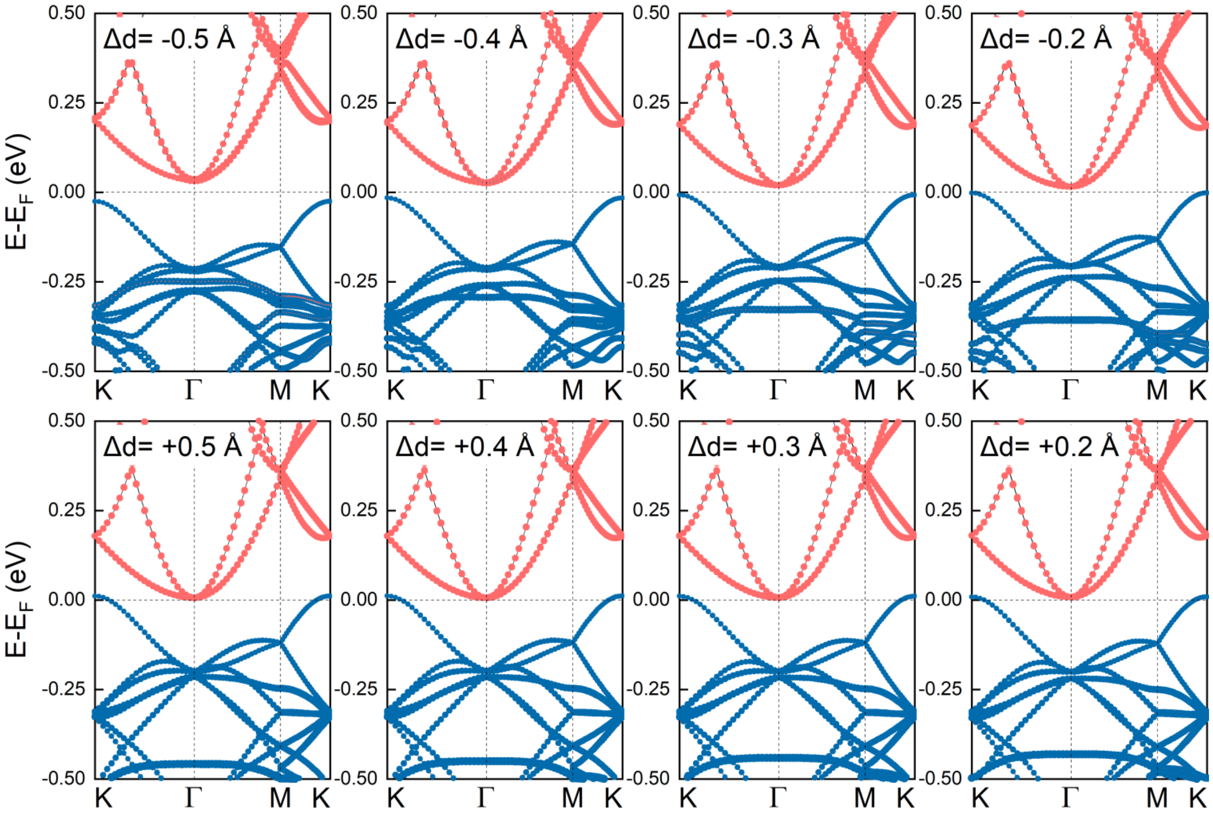

Figure S25: Electronic band structures of low-strained $MoTe_2/ZrS_2$ heterobilayers with rotated layers under out-of-plane strains. Blue and orange lines refer to contributions from $MoTe_2$ and ZrS_2 layers, respectively. Δd corresponds to the interlayer distance variation having as reference the interlayer distance of the ground state structure. SOC is excluded from the calculations.

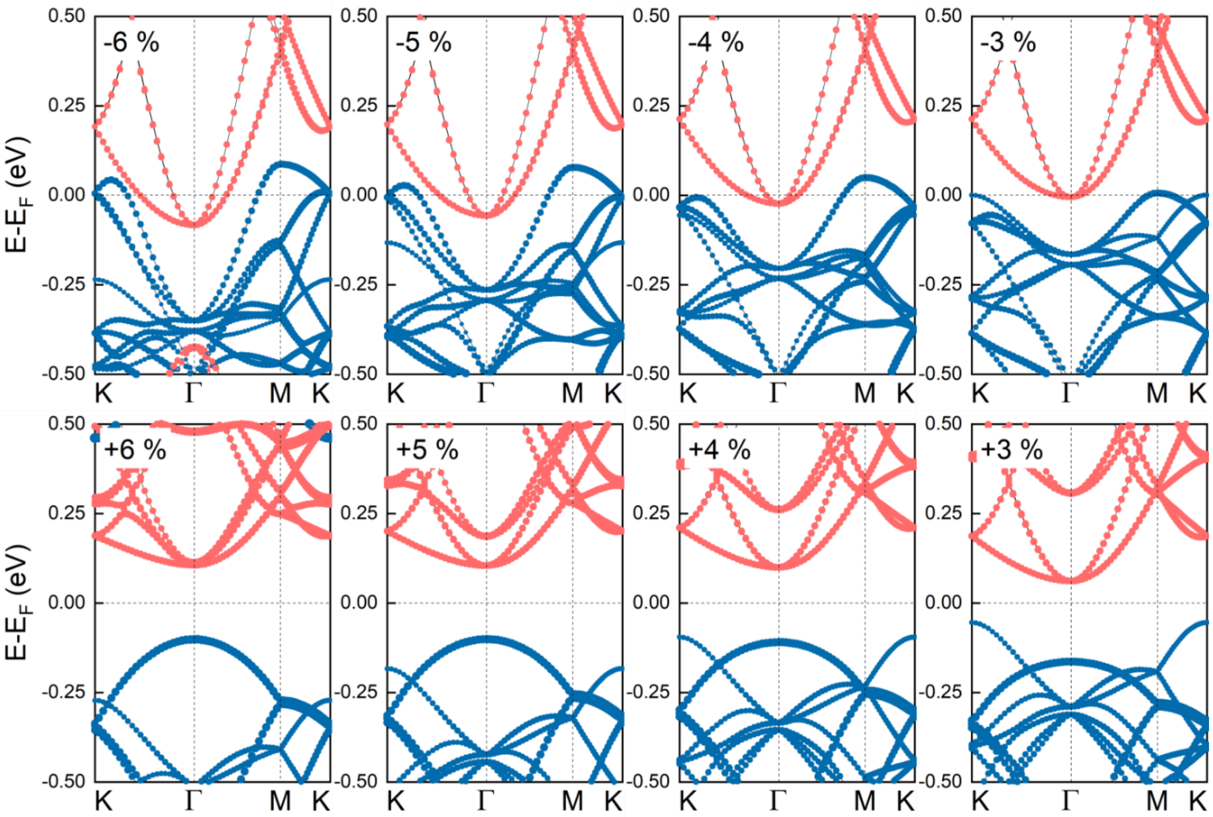

Figure S26: Electronic band structures of low-strained $MoTe_2/ZrS_2$ heterobilayers with rotated layers under in-plane strains. Blue and orange lines refer to contributions from $MoTe_2$ and ZrS_2 layers, respectively. SOC is excluded from the calculations.

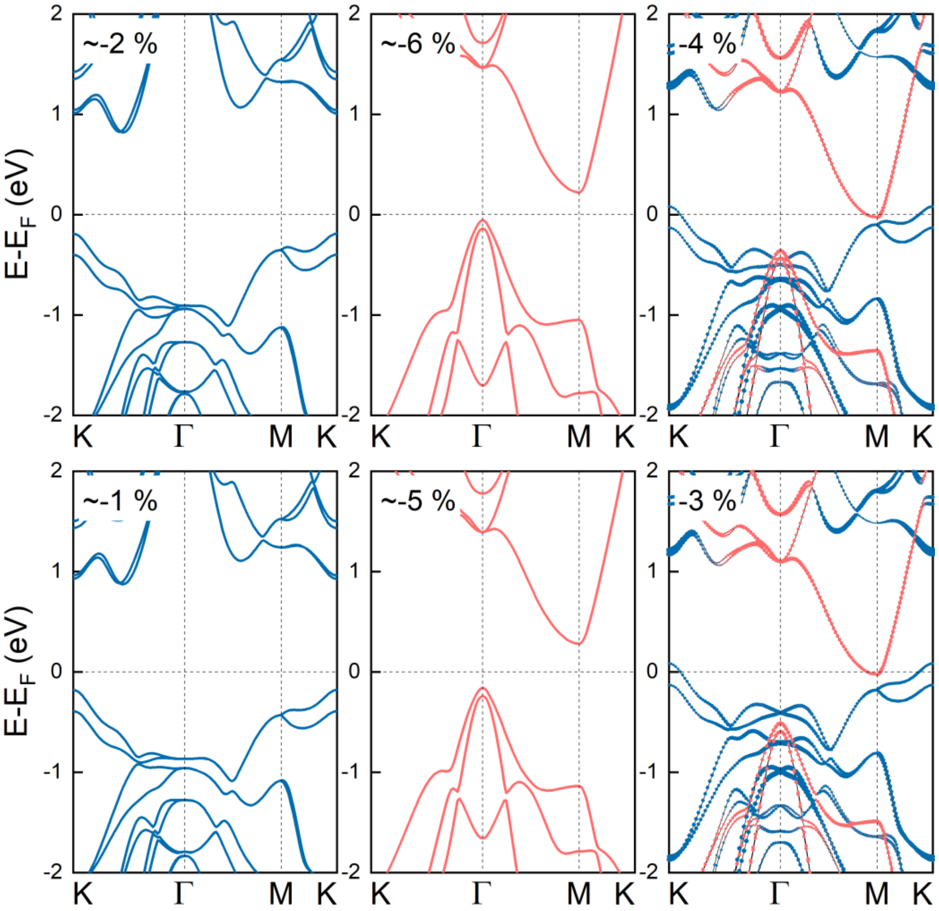

Figure S27: Electronic band structures of (1×1) $MoTe_2$/(1×1) ZrS_2 heterobilayers under in-plane compressive strains and the corresponding band structures of the strained isolated $MoTe_2$ and ZrS_2 monolayers. Blue and orange lines refer to contributions from $MoTe_2$ and ZrS_2 layers, respectively. SOC is included in the calculations.

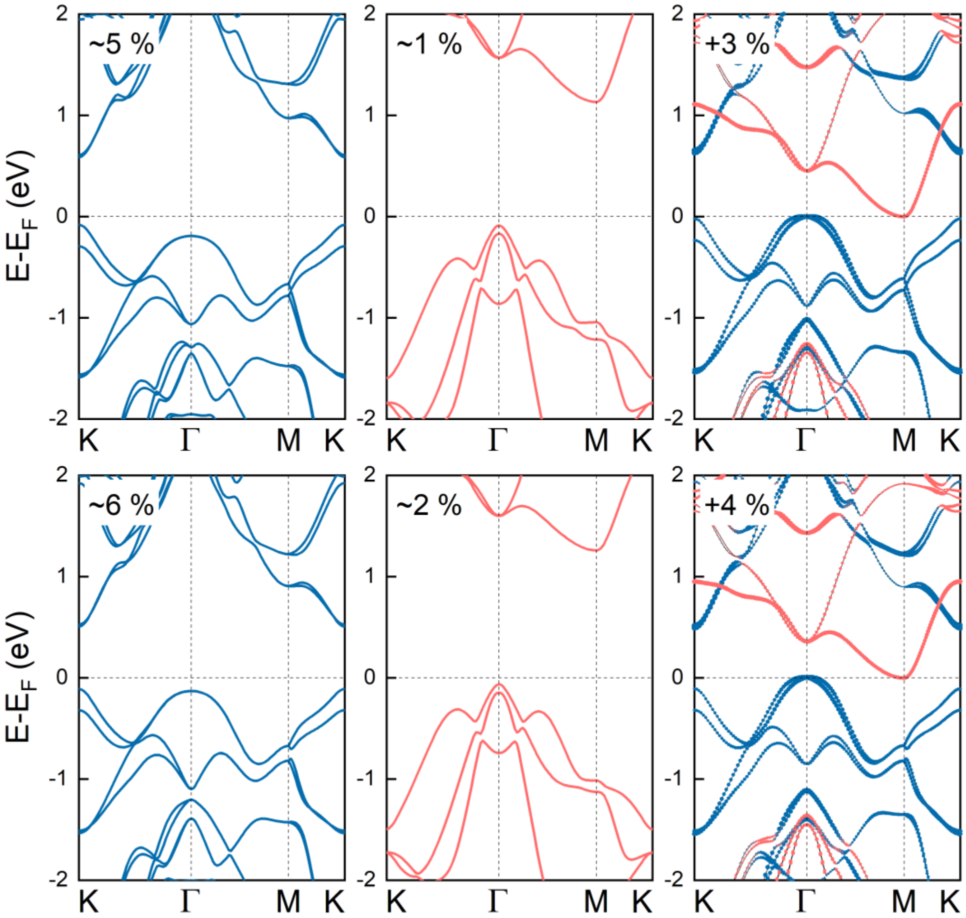

Figure S28: Electronic band structures of (1×1) $MoTe_2$/(1×1) ZrS_2 heterobilayers under in-plane tensile strains and the corresponding band structures of the strained isolated $MoTe_2$ and ZrS_2 monolayers. Blue and orange lines refer to contributions from $MoTe_2$ and ZrS_2 layers, respectively. SOC is included in the calculations.
